# Adhesion of *Rhodococcus* bacteria to solid hydrocarbons and enhanced biodegradation of these compounds

**DOI:** 10.1038/s41598-022-26173-3

**Published:** 2022-12-13

**Authors:** Irina B. Ivshina, Anastasiia V. Krivoruchko, Maria S. Kuyukina, Tatyana A. Peshkur, Colin J. Cunningham

**Affiliations:** 1 Perm Federal Research Centre, 13a Lenin Street, 614990 Perm, Russia; 2 Perm State University, 15 Bukirev Street, 614068 Perm, Russia; 3 Department of Civil and Environmental Engineering, University of Strathclyde, James Weir Building, Level 5, 75 Montrose Street, Glasgow, G11XJ UK

**Keywords:** Environmental biotechnology, Ecophysiology, Microbiology

## Abstract

Adhesive activities of hydrocarbon-oxidizing *Rhodococcus* bacteria towards solid hydrocarbons, effects of adhesion on biodegradation of these compounds by rhodococcal cells and adhesion mechanisms of *Rhodococcus* spp. were studied in this work. It was shown that efficiency of *Rhodococcus* cells’ adhesion to solid *n-*alkanes and polycyclic aromatic hydrocarbons (PAHs) varied from 0.0 to 10.6·10^6^ CFU/cm^2^. *R. erythropolis* IEGM 212 and *R. opacus* IEGM 262 demonstrated the highest (≥ 4.3·10^6^ CFU/cm^2^) adhesion. The percentage biodegradation of solid hydrocarbons (*n-*hexacosane and anthracene as model substrates) by *Rhodococcus* cells was 5 to 60% at a hydrocarbon concentration of 0.2% (w/w) after 9 days and strongly depended on cell adhesive activities towards these compounds (r ≥ 0.71, *p* < 0.05). No strict correlation between the adhesive activities of rhodococcal cells and physicochemical properties of bacteria and hydrocarbons was detected. Roughness of the cell surface was a definitive factor of *Rhodococcus* cell adhesion to solid hydrocarbons. Specific appendages with high adhesion force (≥ 0.6 nN) and elastic modulus (≥ 6 MPa) were found on the surface of *Rhodococcus* cells with high surface roughness. We hypothesized that these appendages participated in the adhesion process.

## Introduction

Solid, or crystalline hydrocarbons, such as long-chain (> 18 carbon atoms) alkanes, polycyclic aromatic hydrocarbons (PAHs), resins, asphaltenes and their complex mixtures (bitumen, oil slimes and oil sludges) are generally considered recalcitrant and poorly biodegradable pollutants with low bioavailability^1–4^. Solid hydrocarbons may be found at petroleum-contaminated sites after evaporation of more volatile components (e.g. C5–C9 alkanes and monoaromatics) and biodegradation of more available hydrocarbons (C10–C18 *n-*alkanes and the volatile and partially water soluble PAH naphthalene), or can originate from industrial wastes or incomplete combustion of organic matter^3,5–8^. A greater understanding of the mechanisms of biodegradation of solid hydrocarbons will improve knowledge of how these compounds undergo natural attenuation and also assist in developing more efficient bioremediation techniques.

It has been previously shown that adhesion of bacterial cells to a hydrocarbon-water interface can enhance biodegradation of hydrocarbons. Growth on and biodegradation of liquid hydrocarbons and their mixtures such as liquid *n-*alkanes (*n-*decane, *n-*tetradecane, and *n-*hexadecane), crude oil, diesel fuel and PAHs (naphthalene, anthracene, phenanthrene, fluorene, fluoranthene, pyrene, etc.) dissolved in non-aqueous phase liquid by bacteria of genera *Acinetobacter*, *Arthrobacter*, *Bacillus*, *Mycobacterium*, *Novoshingobium*, *Pseudomonas*, *Rhodococcus* and by microbial consortia have been described in the literature^9–13^. Several studies have reported bacterial adhesion to solid hydrocarbons and its influence on biodegradation efficacy; these hydrocarbons include PAHs, mainly anthracene and phenanthrene^14–16^. Increased hydrocarbon degradation by adhered cells may be related to minimization of the diffusion path from a hydrocarbon to the cell interior^17^. Additionally, a labile lipophilic mesophase can be formed at the interface between cell and hydrocarbon substrate similar to that detected during oxidation of betulin by *Rhodococcus rhodochrous*. A putative function of the mesophase is slow dissolution of a hydrophobic substrate and its transport to the cell wall^18^. It is postulated that adhesion is not the only factor responsible for biodegradation but adhesion is particularly important when hydrocarbons are not emulsified and their uptake into cells happens as direct contact of cells with hydrocarbons^10^.

Factors of bacterial adhesion to solid abiotic surfaces are physicochemical properties of cells and carriers (hydrophobicity and charge), thermodynamic effect, available surface, and environmental conditions^19,20^. Physicochemistry and thermodynamics of bacterial adhesion are systematized within the frameworks of thermodynamic and extended DLVO theories; however, these theories do not always correctly explain results of the adhesion tests^20–23^. Geometry, topography and roughness of carriers in combination with distribution of physicochemical properties (hydrophobic/hydrophilic and charged/neutral sites) on the carrier surface define the number of available sites for cell attachment, the total contact area, and final number of adhered cells^19,24^. A significant role in adhesion is given to stereospecific interactions relevant to the cell-bound polymers^20,25,26^. An insufficiently studied factor is the cell surface relief. It is known that specialized cell appendages, such as flagella, fimbriae (pili), and specific cytoadhesive nanofibers of e.g., *Acinetobacter* sp. Tol 5, *Caulobacter crescentus*, some Archaea, and *Pseudomonas fluorescens* participate in bacterial adhesion. These appendages have low surface energy, may contain adhesins that facilitate contact between cells and a carrier, provide multilocus binding with significantly changed free energy of adhesion ΔG_adh_, and result in irreversible adhesion^20,23,27,28^. However, many prokaryotes have no pili, flagella and specific cytoadhesive nanofibers but they can have rough cell surface with multiple points of contact and provide multilocus binding followed by tight attachment of cells to the carrier, and this effect is not evaluated. Concerning the specific mechanisms of bacterial adhesion to solid hydrocarbons, they are not fully elucidated in the literature. The dependence of adhesive activities on cell hydrophobicity and zeta potential has been documented, however, these relationships are not fully understood^14,15^. An increase in cell surface roughness is detected during biofilm formation by *Bacillus thuringiensis* on a phenanthrene layer that can evidence the involvement of cell relief and cell appendages in bacterial adhesion to solid hydrocarbons^29^. In addition, it has been suggested that extracellular polymeric substances are involved in the contact between *Pseudomonas putida* and crystalline phenanthrene and fluorene for their degradation^30^.

*Rhodococcus* bacteria (Actinomycetia class) are known degraders of petroleum hydrocarbons able to degrade linear, branched and cyclic alkanes with various chain lengths, mono- and polyaromatic hydrocarbons, and complex hydrocarbon mixtures, such as crude oil, diesel fuel, gasoline, jet fuel, oil slimes, etc.^31–37^. It is known that *Rhodococcus* bacteria can tightly and irreversibly bind to solid surfaces, namely polystyrene and sawdust^24,38^, and adhesion of rhodococci to the oil–water interface is important for degradation of liquid hydrocarbons^39^. Adhesion of rhodococci to solid hydrocarbons is not investigated. The aim of this study was to compare adhesive and oxidizing activities of *Rhodococcus* bacteria towards solid hydrocarbons (long-chain *n-*alkanes and PAHs), to select promising strains for degradation of these compounds and define key factors impacting adhesion of *Rhodococcus* bacteria to solid hydrocarbon substrates.

## Materials and methods

### Reagents and hydrocarbons

All reagents used and Luria–Bertani broth (LB) were > 97% purity and purchased from Sigma-Aldrich. Substrates used for adhesion and biodegradation by *Rhodococcus* cells were five *n-*alkanes with the chain length between 22 and 31 carbon atoms (*n*-docosane, *n*-hexacosane, *n*-octacosane, *n*-nonacosane, and *n*-hentriacontane) and five PAHs with 2 to 5 condensed benzene rings (naphthalene, anthracene, phenanthrene, benzo[a]anthracene, and benzo[a]pyrene). The solubility of hydrocarbons in water and their hydrophobicity in the form of logarithm of *n*-octanol–water partition coefficient, *log*P_O/W_, were the experimental values taken from^40^ or theoretically calculated numbers taken from www.chemspider.com and www.molinspiration.com (Calculation of molecular properties and prediction of bioactivity → draw molecule below → Calculate properties) (Table 1).

**Table 1:** Physicochemical properties of hydrocarbons.

Hydrocarbon	Solubility in water, μmol/L	Hydrophobicity coefficient (*log*P_O/W_)
Naphthalene	236–269	3.5
Phenanthrene	6–10	4.3
Anthracene	0.2–0.5	4.3
Benzo[a]anthracene	0.041–0.061	5.5
Benzo[a]pyrene	0.005–0.019	6.0
*n-*Docosane	0.000001–0.000003	9.7
*n-*Hexacosane	0.00000002–0.000001	10.0
*n-*Octacosane	0.000000002–0.000001	10.1
*n-*Nonacosane	0.0000000007–0.000001	10.2
*n-*Hentriacontane	0.00000000007–0.0000009	10.3

### Bacterial strains and growth conditions

The 82 pure identified non-pathogenic cultures of *Rhodococcus* spp. from the Regional Specialised Collection of Alkanotrophic Microorganisms (acronym IEGM, WDCM # 768, http://www.ckp-rf.ru/usu/73559/, http://www.iegmcol.ru) belonging to *R*. *erythropolis* (14 strains), *R. fascians* (4 strains), *R. jostii* (7 strains), *R. opacus* (9 strains), *R. qingshengii* (1 strain), *R. rhodochrous* (11 strains), *R. ruber* (32 strains), and *Rhodococcus* sp. (4 strains) were used in this study (Table S1). Strains were isolated from various sources (mainly from polluted sites) and able to oxidize crude oil and its components.

Bacteria were grown in Erlenmeyer flasks containing 100 mL of LB on an orbital shaker (160 rpm) at 28 °C for 28–30 h (early stationary phase). Cells were washed twice and resuspended in 0.5% NaCl to the final concentration of ~ 1 × 10^8^ colony-forming units (CFU)/mL that corresponded to OD_600 nm_ = 1.0.

### Adhesive activity tests

Adhesive activities of *Rhodococcus* strains were determined using flat-bottom 96-well polystyrene microplates (Medpolymer, Russia). The microplates were either left untreated or used after modification with hydrocarbons. For modification, individual hydrocarbons were dissolved in acetonitrile at a concentration of 20 mM. When facilitation of hydrocarbon dissolution was required, heating in a water bath at 70 °C was applied. The obtained hydrocarbon solutions were added to microplates at a volume of 300 μL per well. Microplates were then left in a fume cupboard at room temperature for 24 h to allow for the evaporation of acetonitrile. Wells of the modified polystyrene microplates were observed to be completely covered with hydrocarbons (Fig. S1).

*Rhodococcus* cell suspensions in 0.5% NaCl (200 μL per well), transferred onto microplates with modified and unmodified wells, were incubated in a Titramax 1000 incubator (Heidolph Instruments, Germany) at 600 s^−1^ and 28 °C for 48 h. Then, the suspensions were decanted and plates were washed twice with 0.5% NaCl. A 1% (w/v) solution of aqueous crystal violet dye (200 μL per well) was then added onto the microplates. After 20 min at room temperature, the dye solution was removed and the plates were washed twice with 0.5% NaCl. Crystal violet was extracted with the acetone/ethanol mixture (1:4, v/v), and absorbance A_630 nm_ was measured with a Multiscan Ascent photometer (Thermo Electron Corporation, Finland)^38^. A calibration curve between A_630 nm_ and CFU/mL was used to quantify the number of adhered cells (Fig. S2). The adhesive activities were expressed in percentage of attached cells of the initial number of cells in the suspension and as means ± standard deviations of the attached cell number per square unit (CFU/cm^2^).

### Viability of *Rhodococcus* cells

To estimate the viability of attached *Rhodococcus* cells, 200 μL of 0.5% NaCl and 50 μL of a water solution of 0.02% (w/w) iodonitrotetrazolium violet (INT) were added to microplates with washed adhered cells. Intensity of the INT staining, indicating the living and actively respiring cells, was estimated after 2 h of incubation with INT^41^. To screen *Rhodococcus* strains for their ability to grow in the presence of selected long-chain *n-*alkanes and PAHs, polystyrene microplates were filled with minimal medium K (300 μL per well), and hydrocarbons, each dissolved in acetone at a concentration of 20 mM (3 μL per well). Medium K contained (g/L): KH_2_PO_4_—1.0, K_2_HPO_4_—1.0, NaCl—1.0, KNO_3_—1.0; MgSO_4_—0.2, FeCl_3_—0.02, CaCl_2_—0.02, yeast extract—0.05, and trace element solution—1 mL/L (http://www.iegmcol.ru/medium/med08.htmL). Microplates were left at room temperature for 24 h to remove acetone, inoculated with *Rhodococcus* cells (1 × 10^6^ CFU/mL), and incubated at 600 s^−1^, 28 °C for 72 h. After that, 50 μL of 0.02% (w/w) INT was added and intensity of staining was estimated 2 h later. The inoculated medium without hydrocarbons and cell free medium with hydrocarbons were controls, where no color appeared after staining.

### Biodegradation experiments

Biodegradation experiments were performed in 250-mL Erlenmeyer flasks with 100 mL of medium K and 0.2% (w/v) anthracene or *n-*hexacosane at 160 rpm and 28 °C for 9 days. Hydrocarbons were added into medium directly as solid crystals. Initial concentration of *Rhodococcus* cells was 1 × 10^6^ CFU/mL. Residual hydrocarbons were determined gravimetrically^42^ and by gas chromatography with mass spectrometry (GC–MS) after extraction with chloroform. An Agilent 6890 N chromatograph equipped with a quadrupole detector Agilent MSD 5973 N (Agilent Technologies, USA) was used for the GC–MS analysis. A volume of 1 μl of each extract was introduced into an injection port held at 250 °C. The initial oven temperature was 40 °C for 5 min followed by a heating rate of 12 °C/min up to 300 °C, and held at for 10 min. Separation was achieved using a 30 m HP-5MS column with an internal diameter of 0.25 mm and film thickness of 0.25 M (Agilent Technologies, USA) maintained at a constant flow of 1 ml/min of helium. Medium K with hydrocarbons without cells was used as an abiotic control. Cells incubated in the minimal medium with 0.2% (w/v) D-glucose were used as a biotic control.

Interactions of *Rhodococcus* cells with hydrocarbons were studied using a combined microscopic system consisting of an Asylum-MFP-3D-BIO atomic force microscope (AFM) (Asylum Research, USA) and an Olympus FV1000 confocal laser scanning microscope (CLSM) (Olympus Corporation, Japan). A drop (15–20 μL) of cell culture or abiotic control was placed on a cover glass (24 × 50 × 0.15 mm), which was pre-treated with 70% ethanol, mixed with the same volume of a two-component fluorescent dye LIVE/DEAD® *Bac*Light™ Bacterial Viability Kit (Invitrogen, USA) and left at room temperature in darkness for 10–15 min. After staining, the glass was rinsed with deionized water to remove unbound cells and dye. This was followed by CLSM scanning at magnification of × 1000, with scanning rate of 40 nm/pixel and excitation/emission with argon (λ = 488 nm, a 505/525-nm barrier filter) and He–Ne lasers (λ = 543 nm, a 560/660-nm barrier filter) for SYTO 9 and propidium iodide dyes, respectively. CLSM images (0.12 mm × 0.12 mm in size) and resolution of 1600 × 1600 pixels were obtained and analyzed by FV10-ASW 3.1 software (Olympus Corporation, Japan). Cells with green, red and mixed green/red fluorescence were considered as living, dead and unstained/partially damaged cells, respectively. The CLSM images were imported into the AFM software (Igor Pro 6.22A, WaveMetrics, USA), and AFM scanning of the same area was performed. Cells were scanned using AC mode imaging in air at frequency of 0.2 Hz. Silicon cantilevers (AC240TS) without coating, and with resonance frequency of 50–90 kHz, the spring constant of 0.5–4.4 N/m and the radius of 9 nm (Olympus Corporation, Japan) were used.

### Determination of hydrophobicity and zeta potential of *Rhodococcus* cells

Hydrophobicity of rhodococcal cells was determined using methods of microbial adhesion to solvents (MATS), microbial adhesion to hydrocarbons (MATH) and the salt aggregation test (SAT) in accordance with recommendations published elsewhere^43–45^. The polar liquid substrates used for MATS were water, chloroform, diethyl ester, and ethyl acetate, and the nonpolar substrates included *n-*hexane, *n-*decane, and *n-*hexadecane. MATH was done using *n-*hexadecane. For SAT, cells were resuspended in a phosphate buffer supplemented with (NH_4_)_2_SO_4_ at concentrations of 0.2–2.0 M. Cell hydrophobicity was negatively correlated with the (NH_4_)_2_SO_4_ concentration initiating cell aggregation.

The zeta potential of cells was estimated by the dynamic light scattering technique using a ZetaSizer Nano ZS analyzer (Malvern Instruments, UK) and the Malvern ZetaSizer software, v. 2.2 (Malvern Instruments, UK). Washed cells were resuspended in 0.1 M KNO_3_ (pH 7.0), until OD_600 nm_ was 0.2. Measurements were carried out in a U-shaped cuvette with gold-plated electrodes at 25 °C and pH 7.0.

### Studies of the cell surface topography and nanomechanical properties

*Rhodococcus* cells were scanned using AFM in contact mode imaging in liquid (a drop of 0.5% NaCl). Previously, *Rhodococcus* cells were immobilized. For this, drops (15–20 μL) of *Rhodococcus* cell suspension (OD_600 nm_ = 0.5) were placed on cover glasses treated with 0.2% (w/w) polylysine (Sigma-Aldrich). The glasses were air-dried for 30 min, then left at 100% humidity and room temperature in a desiccator with 93% KNO_3_ for 2 days and, finally, rinsed with deionized water^46^. Silicon nitride cantilevers (TR400PB) with Cr/Au coating, resonance frequency of 7–14 kHz, spring constant of 0.01–0.05 N/m, and radius of 42 nm (Olympus Corporation, Japan) were used for scanning. Individual cells were framed and scanned with the maximum resolution of 32 × 32 pixels at a rate of 1 line/s. After scanning the cell surface relief, force mapping was performed for the same cells. Means ± standard deviations of cell surface roughness R_a_ (nm), adhesion force F_a_ (nN), elastic modulus E (MPa), and distribution of these parameters on the cell surface were determined for each strain. For each replicate, three polylysine cover glasses and six cells on each glass were analyzed. Values of R_a_ ≤ 100 nm and outlier values of F_a_ and E were excluded from calculations. R_a_ ≤ 100 nm was frequently detected at cell borders, and we considered these values to be associated with background (surface of polylysine treated cover glasses). Mean ± standard deviation and median for R_a_ on the cell-free polylysine treated cover glasses were 81 ± 50 and 81 nm, respectively.

### Statistics

All experiments were performed in 3–24 replicates. Statistical analysis including determination of the data type distribution, calculation of means ± standard deviations, medians, 25–75%, and correlation coefficients was done using the Statistica (data analysis software system), version 13, TIBCO Software Inc. (2018), http://tibco.com. Differences and correlations were considered statistically significant at *p* < 0.05.

## Results

### Adhesion of *Rhodococcus* bacteria to a reference solid surface (polystyrene)

**Figure 1:**
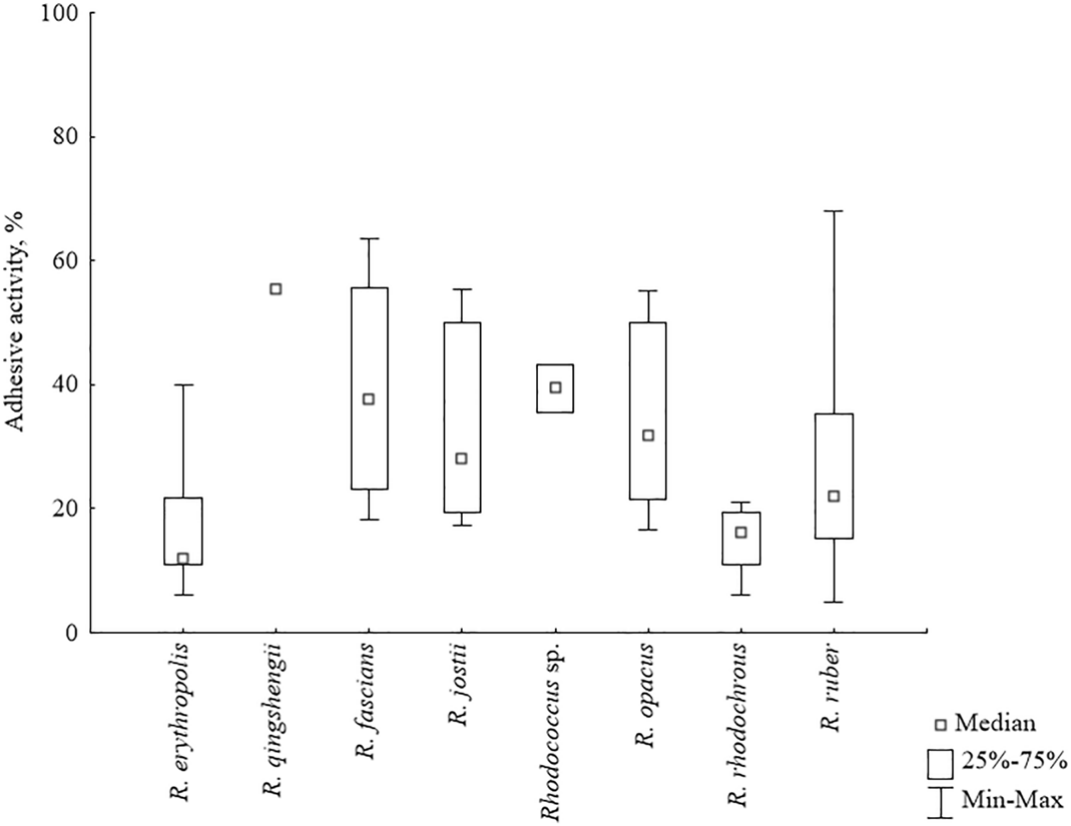
Adhesive activities of *Rhodococcus* strains towards the reference solid surface (polystyrene) depending on the species.

Untreated polystyrene was used as a reference solid surface for *Rhodococcus* cell adhesion. Adhesive activities of *Rhodococcus* strains towards polystyrene grouped by species are shown in Fig. 1. The percentage of adhered rhodococcal cells varied from 6 to 68%, which corresponded to 0.7·10^6^–7.5·10^6^ CFU/cm^2^. No strict correlation between *Rhodococcus* species and their adhesive abilities was revealed. However, *R. rhodochrous* strains had low adhesive activities in a narrow range of 14–21%, and *R. erythropolis* strains adhered to polystyrene relatively weakly (median = 12%).

Based on the screened adhesive activities of *Rhodococcus* to polystyrene, 12 strains from four ecologically significant *Rhodococcus* spp. (*R. erythropolis*, *R. opacus*, *R. rhodochrous*, and *R. ruber*) were selected for experiments on adhesion to and biodegradation of selected C22–C31 *n*-alkanes and PAHs (Table S2). For each species, strains with high, average and low adhesive activities were included in the screening. One strain was *R. qingshengii* IEGM 267 (https://www.ncbi.nlm.nih.gov/assembly/GCF_001900745.1/). This species was described in 2007^47^ and proved to be a synonym of *R. erythropolis* (https://lpsn.dsmz.de/species/rhodococcus-qingshengii). Since the taxonomy of close species such as *R. qingshengii*, *R. baikonurensis*, and *R. erythropolis* is unresolved^48^, we have regarded IEGM 267 as a member of an *R. erythropolis* group.

### Adhesion of *Rhodococcus* strains to solid *n*-alkanes and PAHs

**Figure 2:**
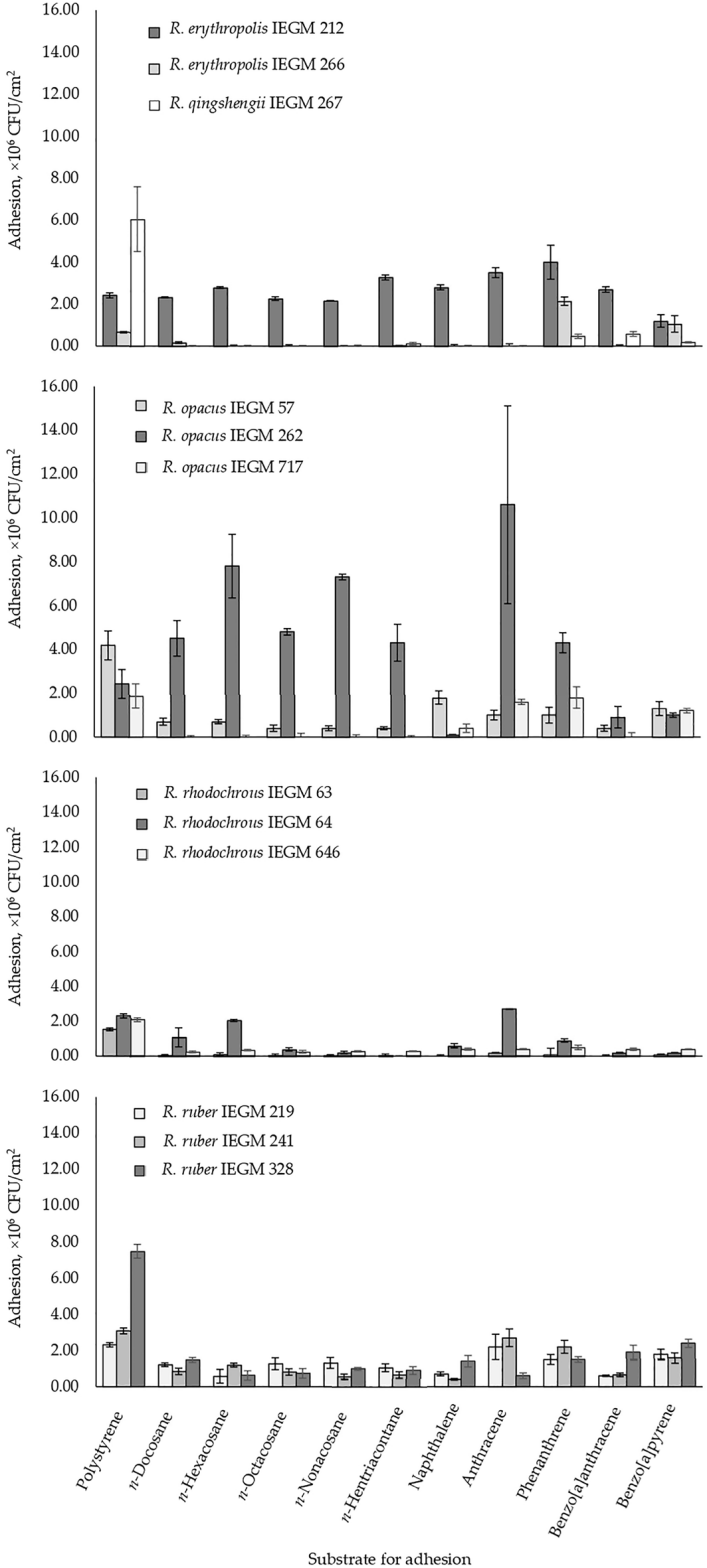
Adhesive activities of selected *Rhodococcus* strains towards C22–C31 *n-*alkanes, PAHs, and the reference solid surface (polystyrene).

*Rhodococcus* strains had various adhesive activities towards solid hydrocarbons ranging from 0.0 to 10.6·10^6^ CFU/cm^2^ (Fig. 2). Again, no evident correlation between *Rhodococcus* species and their adhesive abilities was revealed, although members of *R. rhodochrous* and *R. ruber* adhered to long-chain *n-*alkanes and PAHs with similar low efficiencies (no more than 2.7·10^6^ CFU/cm^2^). While the adhesive activities of *R. erythropolis/R. qingshengii* and *R. opacus* differed by 106–133 times among strains of one species. The highest numbers of adhered cells were 4.3·10^6^–10.6·10^6^ CFU/cm^2^, i.e. 39–96%, and were observed for *R. opacus* IEGM 262, which adhered strongly to almost all hydrocarbons tested, except for naphthalene, benzo[a]anthracene, and benzo[a]pyrene. Adhesion of IEGM 262 to these three hydrocarbons was as low as 0.1·10^6^–1.0·10^6^ CFU/cm^2^. Another strain with high adhesive abilities was *R. erythropolis* IEGM 212. This strain adhered to all hydrocarbons tested with similar efficiencies (1.2·10^6^–4.0·10^6^ CFU/cm^2^). No strict preferences in the adhesion of *Rhodococcus* spp. to hydrocarbons (e.g. to highly available or inaccessible substrates) were documented, however the 3-ring PAHs anthracene and phenanthrene were somewhat preferable substrates as eight strains adhered to these compounds at between 2 and 106 times better than to other hydrocarbons (Fig. 2).

It was shown that the adhesive abilities of most *Rhodococcus* strains towards solid hydrocarbons differed from those towards the reference solid surface (polystyrene). The adhesion efficiency of rhodococci to polystyrene was similar and 2–154 times higher than to solid hydrocarbons. In some cases, *Rhodococcus* strains adhered to polystyrene but were not able to adhere to hydrocarbon crystals, except for *R*. *opacus* IEGM 262 that adhered to hydrocarbons 2–4 times better than to polystyrene (Fig. 2).

### Biodegradation of solid* n*-alkanes and PAHs by *Rhodococcus* spp

In viability tests, it was shown that C22–C31 *n-*alkanes and PAHs at a concentration of 20 mM had no significant toxic effects on *Rhodococcus* bacteria. Adhered cells were viable, and growth of planktonic *Rhodococcus* cells in the presence of all hydrocarbons was observed. We did not evaluate the toxicity of hydrocarbons quantitatively because the aim of these experiments was to ensure that cells remained viable in biodegradation processes. However, it was found that color reaction after INT staining of cells was more intensive in the presence of *n-*alkanes than with PAHs. The most evident growth in the presence of C22–C31 *n-*alkanes was observed for *R. opacus* IEGM 262, *R. rhodochrous* IEGM 64, IEGM 646, *R. ruber* IEGM 241, and IEGM 328 (Fig. S3).

**Figure 3:**
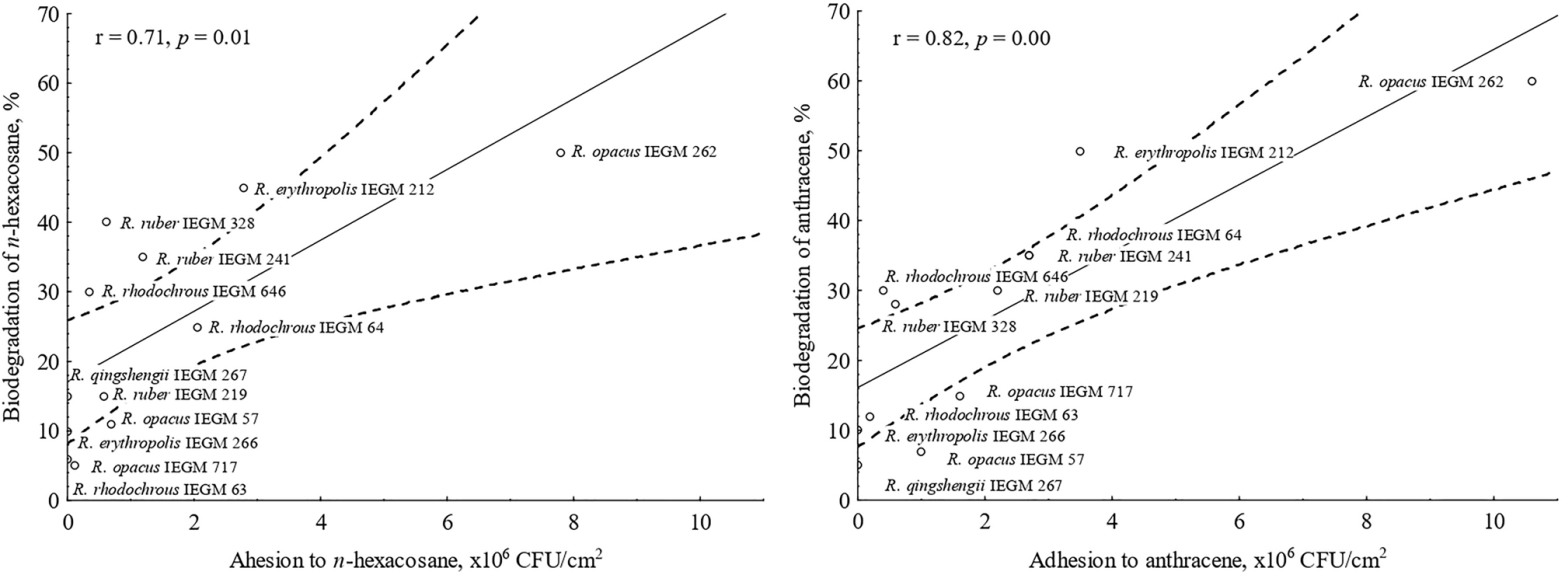
Correlations between the adhesive activities of *Rhodococcus* cells towards *n-*hexacosane and anthracene and biodegradation efficiency of these compounds at their concentration of 0.2% (w/w) after 9 days. Mean values, regression lines and 95% confidence limits are shown; r—Pearson’s correlation coefficient, statistically significant at *p* < 0.05.

In biodegradation experiments, two solid hydrocarbons were used: *n-*hexacosane, a long-chain *n-*alkane with 26 carbon atoms, and anthracene, a middle-weight PAH with three condensed benzene rings. It was revealed that the percentage of *n-*hexacosane and anthracene biodegradation by *Rhodococcus* bacteria varied from 5 to 60% at a hydrocarbon concentration of 0.2% (w/w) after 9 days, as was shown by both gravimetrical and GC–MS analysis. The biodegradation efficiency correlated significantly with adhesive activities of cells towards *n-*hexacosane and anthracene (r ≥ 0.71, *p* < 0.05) (Fig. 3).

**Figure 4:**
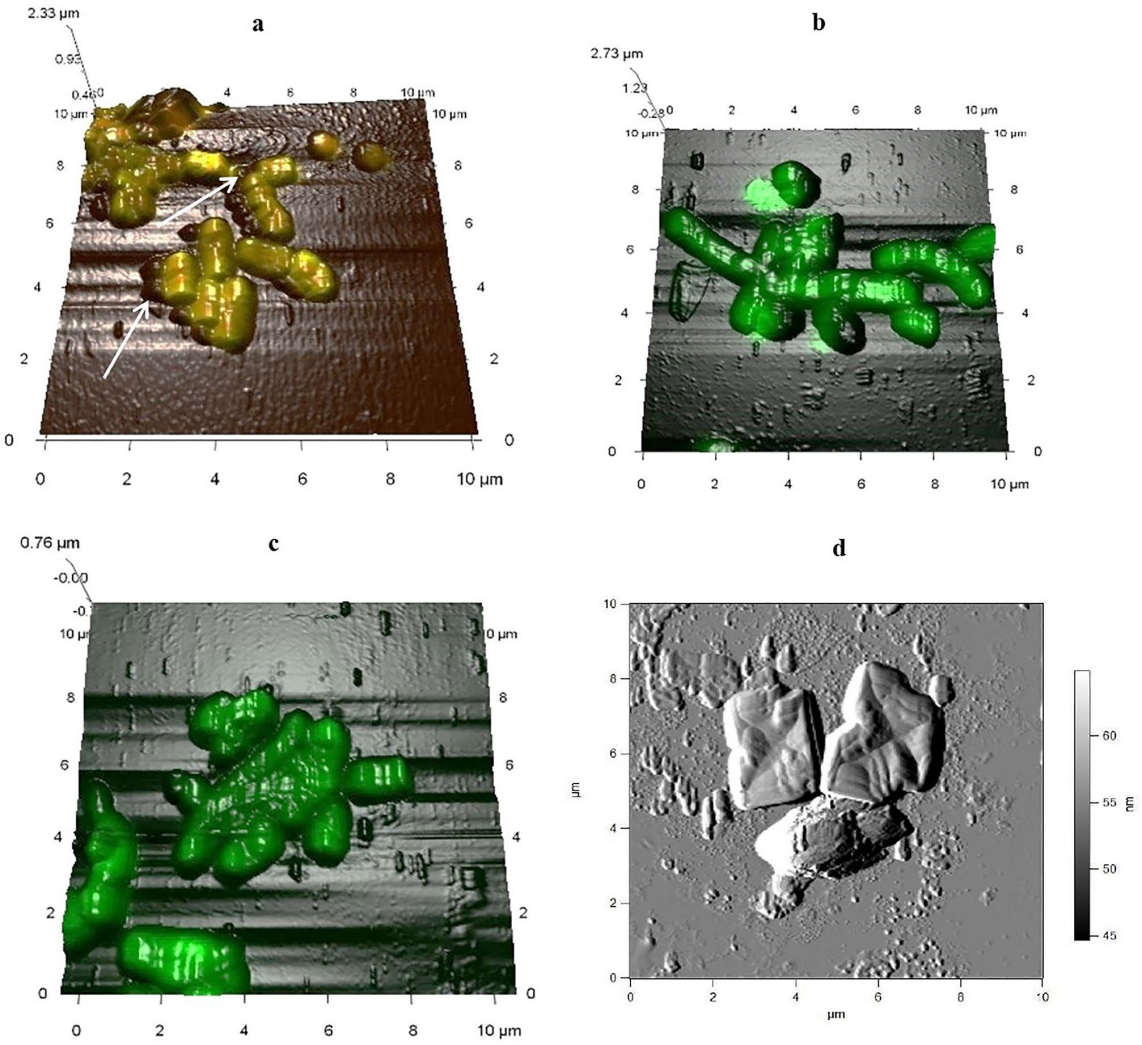
Combined AFM/CLSM images of *Rhodococcus* cells after 9 days of *n-*hexacosane biodegradation. Experimental variants: (**a**) *R. opacus* IEGM 262 grown in the presence of 0.2% (w/w) *n-*hexacosane; (**b**) *R. opacus* IEGM 262 grown in the presence of 0.2% (w/w) D-glucose (biotic control); (**c**) *R. erythropolis* IEGM 212 grown in the presence of 0.2% (w/w) *n-*hexacosane; (**d**) cell-free medium with 0.2% (w/w) *n-*hexacosane (abiotic control). Cells are stained with LIVE/DEAD® *Bac*Light™ Bacterial Viability Kit: green—living cells, mixed green/red—living but partially damaged or unstained cells, black—abiotic objects (hydrocarbon particles). Arrows show hydrocarbon particles.

Using combined AFM/CLSM scanning, it was shown that rhodococci such as *R. erythropolis* IEGM 212 and *R. opacus* IEGM 262 with high adhesive activities towards long-chain *n-*alkanes and PAHs formed aggregates of cells with *n-*hexacosane/anthracene crystals. Because they are not living objects, these crystals were not stained with LIVE/DEAD® *Bac*Light™ Bacterial Viability Kit and were visualized as black 0.5 μm oval objects sticking to cells (Fig. 4). AFM and CLSM scanning confirmed that *Rhodococcus* cells remained viable during biodegradation of solid hydrocarbons indicated by the green color of stained cells. Some strains (e.g. *R. opacus* IEGM 262) were stained green/red (Fig. 4). This mixed coloration was not related to the cell dying and could depend on specific properties of the cell wall, its partial damage or high cell wall thickness hindering transport of dyes into the cell interior^49^.

### Factors of *Rhodococcus* cell adhesion to solid hydrocarbons

No correlation was found between the adhesive and physicochemical properties of *Rhodococcus* cells. Correlation coefficients between adhesive activities of *Rhodococcus* bacteria towards C22–C31 *n-*alkanes and PAHs and cell hydrophobicity determined by the MATH and SAT methods were statistically insignificant. Zeta potential of *Rhodococcus* cells also did not affect their adhesion to polystyrene and solid hydrocarbons (r = − 0.19 to 0.48, *p* ≥ 0.05) (Tables 2, S6). Hydrophobicity and electrokinetic potential are the basic parameters of cells that charactrize their physicochemical properties. As for *Rhodococcus* bacteria, these parameters are known to change proportionally^39,50^. Charged and polar polyelectrolite molecules in the cell wall (exopolysaccharides, proteins, glycolipids, teichoic acids, etc.) determine the negatively charged cell surface. If the numbers of these molecules decrease and lipids increase, the cell wall becomes more hydrophobic and zeta potential shifts to more positive values^25,51^. To illustrate, Table 2 shows two *Rhodococcus* strains with opposite characteristics. *R. erythropolis* IEGM 212 cells were highly hydrophilic (hydrophobicity 19% and 1.4 M according to MATH and SAT, respectively) and negatively charged (zeta potential − 34 mV) compared to *R. rhodochrous* IEGM 64 cells (hydrophobicity 97% and 0.2 M according to MATH and SAT, respectively, and zeta potential − 29 mV) but they adhered to solid hydrocarbons 2–6 times better than cells of IEGM 64. The MATS method, which simultaneously estimated the level of cell hydrophobicity and cell charge, confirmed the lack of dependance between adhesive activities of *Rhodococcus* cells and their physicochemical properties. As shown in Table S7, correlation coefficients between numbers of *Rhodococcus* cells adhered to polystyrene and solid hydrocarbons and their values of adhesion towards MATS-substrates were insignificant (*p* ≥ 0.05). No statistically significant correlation was revealed also between adhesive activities of rhodococcal cells and physicochemical properties of hydrocarbon substrates, such as water solubility, *log*P_O/W_, molecular weight, chain length of *n*-alkanes and the number of condensed benzene rings of PAHs (r ≤ 0.6, *p* ≥ 0.06) (Table S3).

**Table 2:** Physicochemical properties of *Rhodococcus* cells and their influence on adhesion.

Strain	Hydrophobicity of cells		Zeta potential, mV
	MATH, %	SAT, M (NH_4_)_2_SO_4_	
*R. erythropolis* IEGM 212	19 ± 3	1.4	− 34 ± 1
*R. erythropolis* IEGM 266	28 ± 3	1.4	− 28 ± 1
*R. opacus* IEGM 57	97 ± 7	0.2	− 34 ± 2
*R. opacus* IEGM 262	95 ± 5	0.2	− 21 ± 1
*R. opacus* IEGM 717	58 ± 5	0.8	− 35 ± 1
*R. qingshengii* IEGM 267	95 ± 8	0.2	− 27 ± 2
*R. rhodochrous* IEGM 63	20 ± 2	0.8	− 35 ± 1
*R. rhodochrous* IEGM 64	97 ± 5	0.2	− 29 ± 1
*R. rhodochrous* IEGM 646	87 ± 5	0.4	− 29 ± 1
*R. ruber* IEGM 219	96 ± 5	0.2	− 21 ± 0
*R. ruber* IEGM 241	95 ± 5	0.2	− 29 ± 1
*R. ruber* IEGM 328	85 ± 6	0.2	− 28 ± 1
Correlation with adhesive activities towards C22–C31 *n-*alkanes, PAHs and polystyrene	R_Spearman_ ≤ 0.29^1^, *p* ≥ 0.05	R_Spearman_ ≤ 0.57, *p* ≥ 0.05	R_Spearman_ ≤ 0.48, *p* ≥ 0.05

Parameter	Strain		
	*R. erythropolis* IEGM 212	*R. rhodochrous* IEGM 64	
**An example demonstrating the lack of dependence between adhesive and physicochemical properties of *Rhodococcus* cells**			
Adhesion to naphthalene, × 10^6^ CFU/cm^2^	2.8 ± 0.0	0.6 ± 0.1	
Adhesion to *n-*octacosane, × 10^6^ CFU/cm^2^	2.3 ± 0.0	0.4 ± 0.1	
Adhesion to *n-*nonacosane, × 10^6^ CFU/cm^2^	2.2 ± 0.0	0.2 ± 0.1	
Cell hydrophobicity (according to the MATH), %	19 ± 3	97 ± 5	
Cell hydrophobicity (according to the SAT), M (NH_4_)_2_SO_4_	1.4	0.2	
Zeta potential of cells, mV	− 34 ± 1	− 29 ± 1	

Means ± standard deviations are shown for the MATH and the zeta potential values, and medians are shown for the SAT values.

^1^Absolute values for the correlation coefficients are shown.

**Figure 5:**
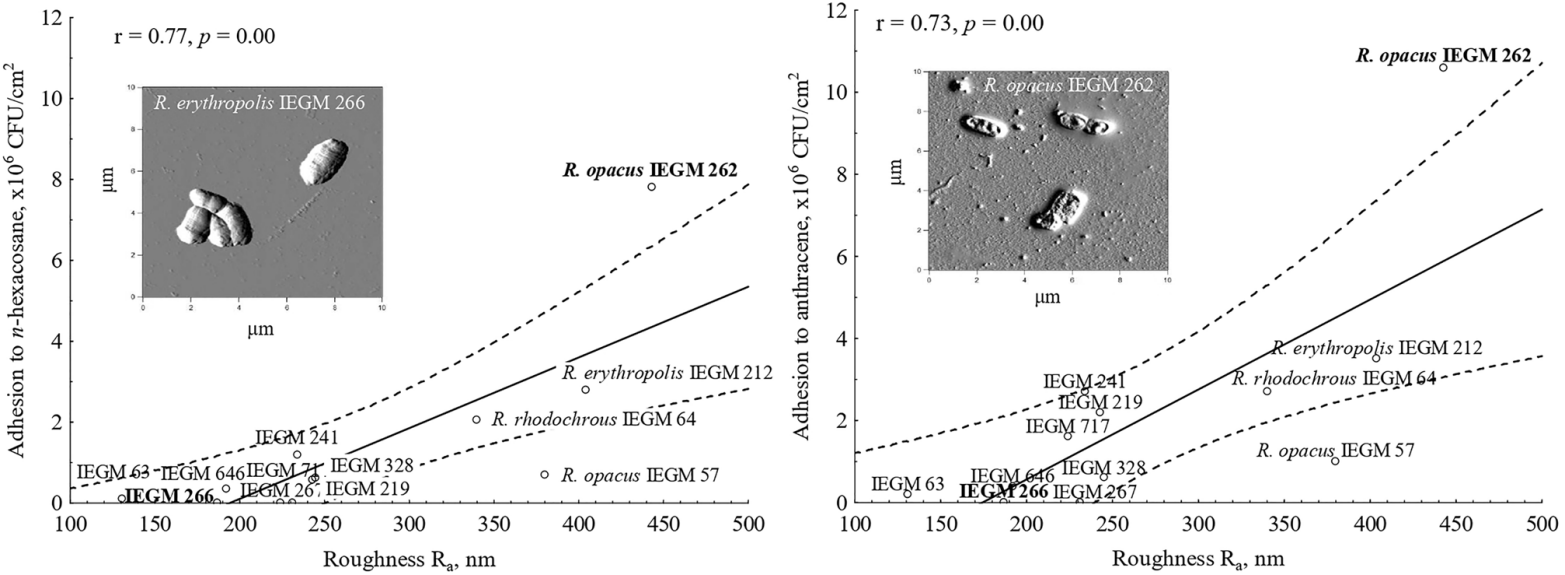
Correlations between surface roughness of *Rhodococcus* cells and their adhesive activities towards *n-*hexadecane and anthracene. Means, regression lines and 95% confidence limits are shown in the graphics; r—Pearson’s correlation coefficient, statistically significant at *p* < 0.05. Cells of strains with high (right) and low (left) levels of roughness are shown in the AFM images.

**Figure 6:**
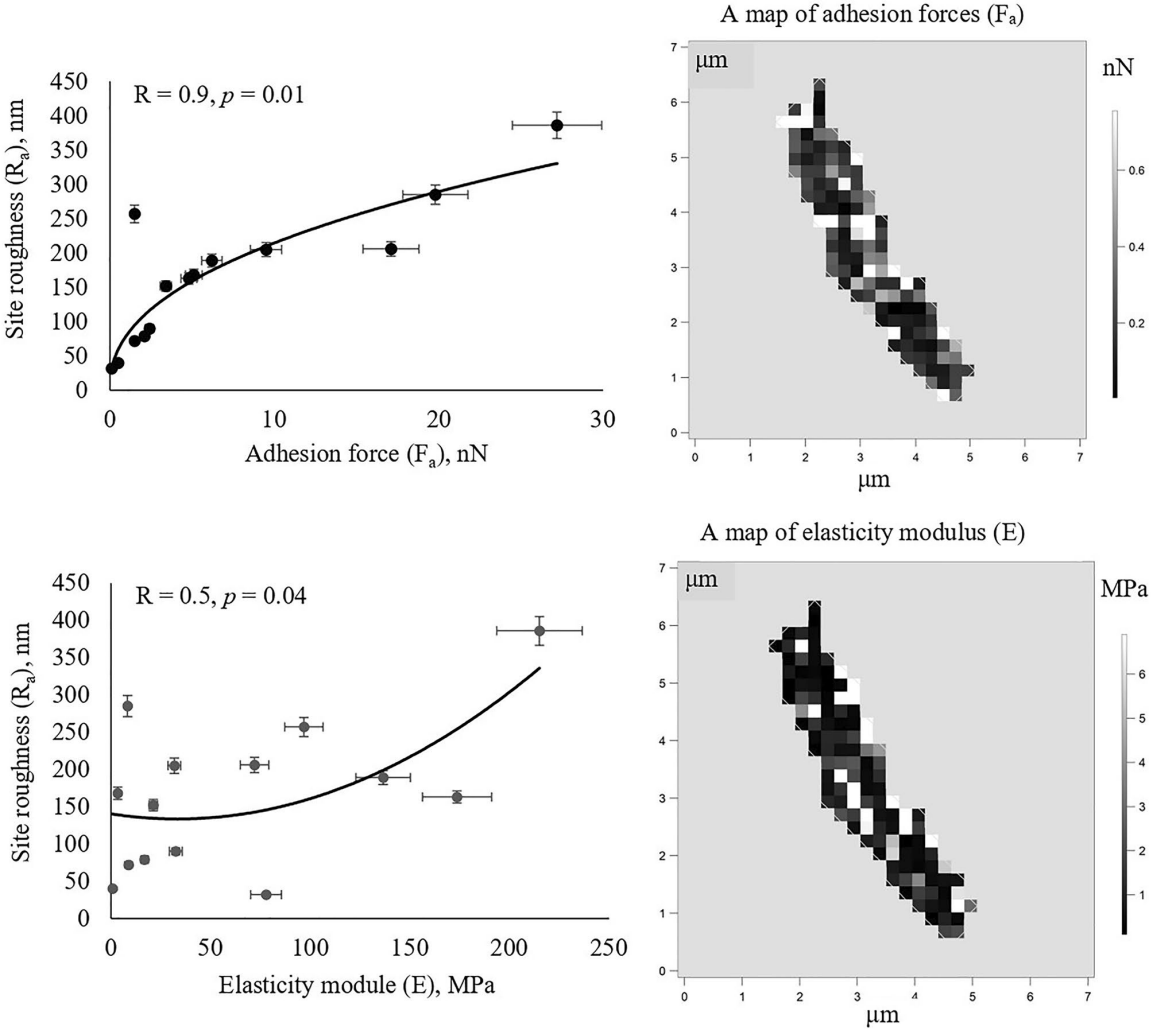
Correlations between nanomechanical parameters (F_a_, E) and roughness (R_a_) of specific cell surface areas, and maps of F_a_ and E distribution values on the cell surface of *R. opacus* IEGM 262. White pixels correspond to cell surface appendages, which are proposed to be specific cytoadhesive structures of *Rhodococcus* spp.

It was shown that adhesive activities of *Rhodococcus* bacteria towards solid *n*-alkanes (*n*-hexacosane) and PAHs (anthracene) depended on cell surface roughness (Fig. 5). Correlation coefficients were 0.77 and 0.73 at *p* = 0.00 for *n*-hexacosane and anthracene respectively. Strains with high (R_a_ = 340–443 nm) and low (R_a_ = 131–245 nm) roughness were distinguished. “Rough” strains were *R. erythropolis* IEGM 212, *R. opacus* IEGM 57, IEGM 262 and *R. rhodochrous* IEGM 64, and the “smooth” group contained other eight strains. The highest level of cell surface roughness was revealed for *R. opacus* IEGM 262 that was able to adhere strongly to all *n*-alkanes and PAHs tested (see Fig. 2). At first approximation, elasticity and adhesiveness of *Rhodococcus* bacteria towards the AFM cantilever did not significantly impact the adhesive activities of cells towards polystyrene and solid hydrocarbons (r = 0.0–0.6, *p* ≥ 0.05). Median values of F_a_ and E for *Rhodococcus* cells are shown in Table S4, and correlation coefficients and *p*-values are shown in Table S5. Only a correlation between the adhesion force of *Rhodococcus* cells and their adhesive activity towards naphthalene was revealed (r = 0.6, *p* = 0.03) (Table S5). However, on the surface of bacterial cells with high roughness, e.g. *R. opacus* IEGM 57, IEGM 262, and *R. rhodochrous* IEGM 64, domains with increased adhesion forces (F_a_ ≥ 0.6 nN) and elasticities (E ≥ 6.0 MPa) were revealed among the most protruding (R_a_ ≥ 200 nm) segments of the cell wall (Fig. 6). Correlation coefficients between F_a_, E and R_a_ of specific areas of the cell surface were positive and amounted to 0.9 at *p* = 0.01 for F_a_ and R_a_ and 0.5 at *p* = 0.04 for E and R_a_, respectively (Fig. 6). These results could be explained by the site-specific chemical composition of these putative appendages and accumulation of distinct compounds there.

## Discussion

In this work, the adhesive activity of *Rhodococcus* bacteria towards solid hydrocarbons in connection with their biodegradation was studied using C22–C31 *n-*alkanes and PAHs as the adhesion substrates. The main conclusions were (1) no strong dependence of adhesion from particular species but strain specificity of the process, (2) relatively low (no more than 2.0·10^6^ CFU/cm^2^, or 18% attached cells) adhesive activities of most *Rhodococcus* strains with only two strains *R. erythropolis* IEGM 212 and *R. opacus* IEGM 262 having high (up to 10.6·10^6^ CFU/cm^2^) adhesive activities towards the tested hydrocarbons, (3) no well-defined substrate specificity, however, some preferences were revealed towards anthracene and phenanthrene, and (4) adhesive activities of many *Rhodococcus* strains towards solid hydrocarbons and the model solid surface (polystyrene) were different.

Rhodococci were able to grow in the presence of all solid hydrocarbons added into the culture medium in a dispersed form (dissolved in a solvent). *Rhodococcus* cells grew more intensively in the presence of dispersed long-chain *n-*alkanes, rather than dispersed PAHs (Fig. S3). Solid *n-*alkanes are not toxic compounds, while PAHs are priority pollutants with confirmed toxicity^52^. Moreover, solubility of PAHs in water is 3–13 orders of magnitude higher than that of C22–C31 *n-*alkanes (Table 1). On the one hand, it makes PAHs more bioavailable and facilitates their degradation by *Rhodococcus* cells. On the other hand, toxic PAHs are more easily transported to the cell interior and also interact with cell walls and membranes in a more destructive way. On the contrary, *n-*alkanes are 2–3 times more hydrophobic than PAHs (Table 1) that could mediate adhesion and make these compounds more available for lipophilic *Rhodococcus* cells even in the dispersed form.

Biodegradation experiments were performed using hydrocarbon crystals. It was shown that adhered *Rhodococcus* bacteria degraded solid hydrocarbons more efficiently. As mentioned above, adhesion is required for the tight bound and close contact between bacterial cells and a hydrocarbon substrate to reduce a diffusion path between them. Interestingly, rhodococci did not interact with large hydrocarbon crystals visualized in AFM images of abiotic controls, such as lamellas 2 × 2 μm in size (Fig. 4). Attachment to smaller particles could give certain advantages to cells since one cell could contact with several small particles, and cells could surround them. It would result in increased contact area and an enhanced rate of degradation. *Rhodococcus* bacteria with low adhesive and hydrocarbon-oxidizing activities (we referred to them as “non-adhesive” strains), for example *R. qingshengii* IEGM 267, did not contact with large *n-*hexacosane and anthracene particles (Fig. 5), but grew in the presence of these dispersed compounds. We speculated that it was related to the contact of cells with a fraction of highly dispersed hydrocarbon crystals, which were not detected by AFM at the used resolution. Probably, this fraction was numerically insignificant that could explain lower rates of hydrocarbon biodegradation. It should be also noted that large *n-*hexacosane and anthracene lamellas were not observed in growing cell cultures. We hypothesized that it could resulted from *Rhodococcus* cell growth, which led to degradation of lamellas.

Adhesion and biodegradation experiments resulted in selection of *R. erythropolis* IEGM 212 and *R. opacus* IEGM 262 as the most promising solid hydrocarbon-degrading strains. Other candidates for bioremediation applications were *R. ruber* strains, *R. opacus* IEGM 57 and IEGM 717 and *R. erythropolis* IEGM 266. These strains, along with IEGM 212 and IEGM 262, showed relatively high, 1.0·10^6^–2.4·10^6^ CFU/cm^2^, adhesive activities towards benzo[a]pyrene (Fig. 2). This 5-ring recalcitrant PAH is the major indicator compound for PAH-contamination. It is a toxic, cancerogenic, mutagenic and teratogenic agent, which is tightly bound to soil particles and bottom sediments, hardly removed from the environment and shows various adverse effects on biotic communities^52–55^. Concentration of benzo[a]pyrene ≥ 0.02 mg/kg soil is considered to be hazardous^52^. *R. rhodochrous* strains had the lowest adhesion potential, and apparently were less suitable for adhesion and further application as degraders of solid hydrocarbons.

A significant part of this work was devoted to studying mechanisms of adhesion. The obtained results demonstrated that physicochemical interactions between *Rhodococcus* cells and liquid hydrocarbon droplets as a result of hydrophobic and electrostatic forces^20^ were apparently not pivotal to adhesion of rhodococci to solid hydrocarbons, namely long-chain *n*-alkanes and PAHs, and polystyrene. According to published estimates of surface energy components^44,56^, polystyrene is more hydrophobic (γ^LW^ = 36.0 mJ/m^2^) than hydrocarbons (γ^LW^ = 18.4–27.7 mJ/m^2^), which could explain better adhesion of some *Rhodococcus* strains to untreated polystyrene microplates in comparison with microplates treated with solid hydrocarbons. However, electrostatic forces appeared to be more involved in adhesion of bacterial cells to polystyrene (γ^–^ = 7.6 mJ/m^2^, γ^+^  = 0.02 mJ/m^2^) than to hydrocarbons (γ^–^ = 0.0 mJ/m^2^, γ^+^  = 0.0 mJ/m^2^) with probable repulsion between negatively charged cells and polystyrene^23,56,57^, and lower adhesion of rhodococci to polystyrene should be expected. The lack of correlation between hydrophobic properties of bacterial cells and their adhesive activities towards solid substrates is not unique and has been described in literature^14,20,22^. Apparently, other factors were important for adhesion of rhodococci.

The influence of the cell surface relief on adhesion of nonpathogenic *Rhodococcus* bacteria to solid hydrocarbons was first shown in this work. Earlier, the type IVb cytoadhesive pili from the subfamily of Flp-pili being the result of a horizontal gene transfer and providing adhesion to mammal epithelial cells and macrophages were described in opportunistic *R. equi*^58^. Bridge-like structures consisting of molecules of the titan-binding protein TiBP and providing adhesion to TiO_2_ were vizualized on cells of nonpathogenic *R. ruber* GIN1^59^. In this work, putative cytoadhesive structures were revealed on *Rhodococcus* cells having high roughness (≥ 200 nm), adhesion force (≥ 0.6 nN) and elastic modulus (≥ 6 MPa). High adhesiveness of these structures to the AFM cantilever most likely occurred as a result of specific chemical composition and accumulation of adhesins. These appendages may contain lipids, whilst polysaccharides or specific proteins may not be excluded. For example, it was shown that the adhesion force of the AFM cantilever to a lipid bilayer and mycolic acids was six times greater than to proteins and polylysine and amounted to 3 nN^21,60^; and F_a_ of the cantilever to proteins and polysaccharides varied between 0.040 and 2.500^61,62^. In this work, the adhesion force values of the AFM cantilever to putative *Rhodococcus* cell appendages were similar to those documented for lipids and exceeded 2–10 times the F_a_ values for polylysine-covered glasses (F_a_ median = 0.785 nN) and this could suggest lipid accumulation in these structures. High elasticity could be explained by high density of the cell wall material and putative adhesive compounds constituting cell surface appendages. A similar effect was shown for agarose gel films microspotted with polylysine aqueous solution. The thickness and density of agarose in polylysine-rich sites were determined to be higher than in sites without polylysine, and following a 1.5–2.0-fold increase of elasticity modulus was observed in polylysine-rich sites^62^. Interestingly, putative cytoadhesive appendages occupied about 20% of the surface of *Rhodococcus* cells (white pixels with F_a_ ≥ 0.6 nN and E ≥ 6.0 MPa in Fig. 6). This could explain the lack of dependency between average values of elasticity and adhesion force of bacterial cells and their adhesive activities towards soild hydrocarbons and polystyrene.

Another factor of *Rhodococcus* cell adhesion to solid hydrocarbons could be surface roughness of hydrocarbons. We assumed though that this factor, along with physicochemical properties of hydrocarbons, was less significant than bacterial surface properties. Adhesion of *Rhodococcus* cells did not depend on substrates, although some preferences towards PAHs (anthracene and phenanthrene) were revealed. Figure S1 shows that PAHs formed a rougher and less uniform coating on the surface of polystyrene microplates compared to C22–C31 *n-*alkanes. Increased roughness of PAH films could explain better adhesion of rhodococci to anthracene and phenanthrene. On the other hand, wells of unmodified polystyrene microplates had a very smooth surface; nevertheless, *Rhodococcus* cells adhered to them with the same or better efficiency than to microplates covered with hydrocarbons; and only *R*. *opacus* IEGM 262 adhered to hydrocarbon films 2–4 times better than to unmodified polystyrene. Moreover, the effects of roughness seem to be different in adhesion tests using polystyrene microplates treated with hydrocarbons and in biodegradation experiments, where hydrocarbon crystals of various sizes were suspended in the growth medium. As seen from Fig. 4d, freely floating crystals had relatively smooth surface without pronounced irregularities, while hydrocarbon films on polystyrene microplates, in particular PAH films, were clearly rougher (Fig. S1). Presumably, the produced microrelief could slow down the rate of cell migration, lead to cell trapping between surface irregularities, impact the entire contact area, and facilitate cell adhesion compared to floating crystals. Nevertheless, biodegradation of solid hydrocarbons by *Rhodococcus* strains strongly depended on cell adhesion to hydrocarbon films. Some specific or stereospecific interactions between *Rhodococcus* cells and hydrocarbons may occur. There is some evidence in literature: *R. ruber* GIN1 with the protein adhesin TiBP binds specifically to TiO_2_^59^; contraction or extension of exopolysaccharides and pili on the surface of bacterial cells leads to their steric attraction or repulsion, affects the density of binding sites and magnitude of the adhesion force^21,25^; and the nanoscopic deformation of the bacterial cell wall detected at cell approaching the carrier increases the contact area and changes membrane surface tension and intensity of physicochemical interactions^20^. Appendages on the surface of *Rhodococcus* cells can theoretically participate in some steric interactions with solid hydrocarbons. Moreover, it is known that hydrocarbon molecules, particularly PAHs, can be arranged in space as packings of various forms^63,64^, and at nanoscale, it can determine steric conformity with cell wall ultrastructures. Some divergencies between the results of MATH and SAT (Table 2) characterizing distribution of hydrophobic domains on the cell surface^43^ may suggest similar non-uniform and mosaic distribution of cell binding sites on the surface of hydrocarbon coatings (e.g. PAH coatings). Interactions between biotic and abiotic surfaces of complex structures can complicate analyzing the bacterial adhesion factors. Such sophisticated mechanisms of *Rhodococcus* cell adhesion to solid hydrocarbons should be further studied to provide better understanding of different efficiencies in their biodegradation. For complete understanding mechanisms of *Rhodococcus* cell adhesion, molecular identification of adhesins and determination of chemical composition of cytoadhesive structures revealed in this study is required, and relevant tests will be done as a separate work in future.

## Conclusion

The data obtained in this study increased the insight into the mechanisms of microbial degradation of solid hydrocarbons such as long-chain alkanes and PAHs and can be applied to heavy fractions of crude oil and solid products of petroleum refinery (e.g. wax, asphalt, and petroleum coke). Using various *Rhodococcus* species, it was shown that adhesion of degrading bacteria to solid hydrocarbons was an important factor for efficient biodegradation of these compounds; however, this does not exclude the influence of other factors, for example, specific oxidizing enzymes. Ability of bacterial strains to adhere to solid hydrocarbons can be used when selecting them as promising agents for bioremediation. In this work, two strains *R. erythropolis* IEGM 212 and *R. opacus* IEGM 262 were selected. They strongly (1.2·10^6^–10.6·10^6^ CFU/cm^2^, or 11–96%) adhered to C22–C31 *n-*alkanes and PAHs with 2–5 benzene rings and oxidized model hydrocarbons, *n-*hexacosane and anthracene, with 45–60% efficiency at a hydrocarbon concentration of 0.2% (w/w) in 9 days. These strains may be recommended for application in heavily contaminated sites with weathered hydrocarbons or in PAH-polluted sites, and may be helpful for development of microbial associations for more effective degradation of complex hydrocarbon mixtures. Novel results were obtained about the adhesion mechanisms. It was revealed that cell surface roughness is the factor determining adhesive activities of *Rhodococcus* bacteria towards solid hydrocarbons. The specific cell wall structures revealed on the surface of *Rhodococcus* cells with high adhesiveness, *R. opacus* IEGM 262 in particular, most probably containing putative adhesins, could play an important role in adhesion of *Rhodococcus* bacteria to solid hydrocarbons.

## Supplementary Information

Supplementary Information.

## Data Availability

The data generated during and/or analyzed during the current study are available from the corresponding author on reasonable request.
