# Supplementary material for: Adhesion of Rhodococcus bacteria to solid hydrocarbons and enhanced biodegradation of these compounds
Source: Sci Rep. 2022 Dec 13;12:21559. doi: 10.1038/s41598-022-26173-3 (PMC9748138; doi:10.1038/s41598-022-26173-3)
Supplement: Supplementary file 1 — Supplementary Information. [file 41598_2022_26173_MOESM1_ESM.pdf]

Table S1 – *Rhodococcus* strains (http://www.iegmcol.ru)

Strain	Isolation source	Public accession
R. erythropolis		
IEGM 20	Oil-polluted soil, Ukraine	http://www.iegmcol.ru/strains/rhodoc/eryth/r_eryth20.html
IEGM 185	Water, Kama reservoir, Perm krai, Russia	http://www.iegmcol.ru/strains/rhodoc/eryth/r_eryth185.html
IEGM 186	Water, Kama reservoir, Perm krai, Russia	http://www.iegmcol.ru/strains/rhodoc/eryth/r_eryth186.html
IEGM 188	Oil-polluted bottom sediments, the Ostjatski Zhivets river, Tyumen region, Russia	http://www.iegmcol.ru/strains/rhodoc/eryth/r_eryth188.html
IEGM 192	Oil-polluted bottom sediments, Bezjimyannoe lake, Tyumen region, Russia	http://www.iegmcol.ru/strains/rhodoc/eryth/r_eryth192.html
IEGM 212	Sewage, Kharbin, China	http://www.iegmcol.ru/strains/rhodoc/eryth/r_eryth212.html
IEGM 265	Oil-polluted soil, oil-extracting enterprise, Perm krai, Russia	http://www.iegmcol.ru/strains/rhodoc/eryth/r_eryth265.html
IEGM 266	Oil-polluted soil, oil-extracting enterprise, Perm krai, Russia	http://www.iegmcol.ru/strains/rhodoc/eryth/r_eryth266.html
IEGM 268	Oil-polluted soil, oil-extracting enterprise, Perm krai, Russia	http://www.iegmcol.ru/strains/rhodoc/eryth/r_eryth268.html
IEGM 269	Oil-polluted soil, oil-extracting enterprise, Perm krai, Russia	http://www.iegmcol.ru/strains/rhodoc/eryth/r_eryth269.html
IEGM 271	Oil-polluted soil, oil-extracting enterprise, Perm krai, Russia	http://www.iegmcol.ru/strains/rhodoc/eryth/r_eryth271.html
IEGM 487	Water, the Baykal Lake, Irkutsk region, Russia	http://www.iegmcol.ru/strains/rhodoc/eryth/r_eryth487.html, GenBank#KF547999
IEGM 708	Oil-shale from settling pit, Polazna oil-extracting enterprise, Perm krai, Russia	http://www.iegmcol.ru/strains/rhodoc/eryth/r_eryth708.html, GenBank#KY194798
IEGM 1189	Water, Tyumen region, Russia	http://www.iegmcol.ru/strains/rhodoc/eryth/r_eryth1189.html, GenBank#MG645202
R. fascians		
IEGM 34	Carp skin	http://www.iegmcol.ru/strains/rhodoc/fascians/r_fasc34.html
IEGM 39	Stratal water, oilfield, Perm krai, Russia	http://www.iegmcol.ru/strains/rhodoc/fascians/r_fasc39.html
IEGM 170	Snow carpet, Polasna oil-extracting enterprise, Perm krai,	http://www.iegmcol.ru/strains/rhodoc/fascians/r_fasc170.html

	Russia	
IEGM 278	River water, Tyumen region, Russia	http://www.iegmcol.ru/strains/rhodoc/fascians/r_fasc278.html
R. jostii		
IEGM 28	Soil, the bank of the pond, Dnepropetrovsk region, Ukraine	http://www.iegmcol.ru/strains/rhodoc/jostii/r_jostii28.html, GenBank#MG912559
IEGM 29	Oil-polluted soil, oil-gas field, Poltava region, Ukraine	http://www.iegmcol.ru/strains/rhodoc/jostii/r_jostii29.html, GenBank#MG912570
IEGM 31	Oil-polluted soil, oil-gas field, Poltava region, Ukraine	http://www.iegmcol.ru/strains/rhodoc/jostii/r_jostii31.html, GenBank#MG912563
IEGM 32	Soil, field, Poltava region, Ukraine	http://www.iegmcol.ru/strains/rhodoc/jostii/r_jostii32.html, GenBank#MG912560
IEGM 33	Oil-polluted soil, oil-gas field, Ivano-Frankovsk region, Ukraine	http://www.iegmcol.ru/strains/rhodoc/jostii/r_jostii33.html
IEGM 60	Oil-polluted soil, oilfield, Ukraine	http://www.iegmcol.ru/strains/rhodoc/jostii/r_jostii60.html, GenBank#MG607376
IEGM 68	Soil, Polasna oil-extracting enterprise, Perm krai, Russia	http://www.iegmcol.ru/strains/rhodoc/jostii/r_jostii68.html, GenBank#MG912561
R. opacus		
IEGM 56	Soil, windbreak, Kherson region, Ukraine	http://www.iegmcol.ru/strains/rhodoc/opac/r_opac56.html
IEGM 57	Oil-polluted soil, Ukraine	http://www.iegmcol.ru/strains/rhodoc/opac/r_opac57.html
IEGM 59	No information	http://www.iegmcol.ru/strains/rhodoc/opac/r_opac59.html
IEGM 246	Soil, lavsan (polyether fibre) production, Belarus	http://www.iegmcol.ru/strains/rhodoc/opac/r_opac246.html, GenBank#MG607380
IEGM 261	Soil, lavsan (polyether fibre) production, Belarus	http://www.iegmcol.ru/strains/rhodoc/opac/r_opac261.html
IEGM 262	Soil, lavsan (polyether fibre) production, Belarus	http://www.iegmcol.ru/strains/rhodoc/opac/r_opac262.html
IEGM 716^{T}	Municipal gasworks defective pipe, UK	http://www.iegmcol.ru/strains/rhodoc/opac/r_opac716t.html
IEGM 717	Soil	http://www.iegmcol.ru/strains/rhodoc/opac/r_opac717.html
IEGM 1157	Soil, Perm, Perm krai, Russia	http://www.iegmcol.ru/strains/rhodoc/opac/r_opac1157.html
R. qingshengii		
IEGM 267	Oil-polluted soil oil-extracting enterprise, Perm krai, Russia	http://www.iegmcol.ru/strains/rhodoc/qingsh/r_qingsh267.html, DDBJ/ENA/GenBank acc. no MRBQ01000001-MRBQ01000231

R. rhodochrous		
IEGM 63	Oil-polluted soil, Ukraine	http://www.iegmcol.ru/strains/rhodoc/rhodoch/r_rhod63.html
IEGM 64	No information	http://www.iegmcol.ru/strains/rhodoc/rhodoch/r_rhod64.html
IEGM 66	No information	http://www.iegmcol.ru/strains/rhodoc/rhodoch/r_rhod66.html, DDBJ/ENA/GenBank acc. no JAJNDE010000001-JAJNDE010000099
IEGM 67	Soil, UK	http://www.iegmcol.ru/strains/rhodoc/rhodoch/r_rhod67.html
IEGM 608	Water, Berezniki, Perm krai, Russia	http://www.iegmcol.ru/strains/rhodoc/rhodoch/r_rhod608.html, GenBank#KY194859
IEGM 632	Water, Berezniki, Perm krai, Russia	http://www.iegmcol.ru/strains/rhodoc/rhodoch/r_rhod632.html
IEGM 639	Snow, oilfield, Perm krai, Russia	http://www.iegmcol.ru/strains/rhodoc/rhodoch/r_rhod639.html
IEGM 646	Water, Berezniki, Perm krai, Russia	http://www.iegmcol.ru/strains/rhodoc/rhodoch/r_rhod646.html
IEGM 647	Oil-polluted water, Mezhevskoe oilfield, Perm krai, Russia	http://www.iegmcol.ru/strains/rhodoc/rhodoch/r_rhod647.html, GenBank#MG607375
IEGM 1137	Oil-polluted soil, Solikamsk, Perm krai, Russia	http://www.iegmcol.ru/strains/rhodoc/rhodoch/r_rhod1137.html
IEGM 1138	Oil-polluted soil, Solikamsk, Perm krai, Russia	http://www.iegmcol.ru/strains/rhodoc/rhodoch/r_rhod1138.html
R. ruber		
IEGM 65	Water, Mississipi river, USA	http://www.iegmcol.ru/strains/rhodoc/ruber/r_ruber65.html
IEGM 73	Ground water, Masuninskoe oilfield outline zone, Perm krai, Russia	http://www.iegmcol.ru/strains/rhodoc/ruber/r_ruber73.html
IEGM 76	Snow, oilfield outline zone, Perm krai, Russia	http://www.iegmcol.ru/strains/rhodoc/ruber/r_ruber76.html
IEGM 77	Water, spring, oilfield outline zone, Perm krai, Russia	http://www.iegmcol.ru/strains/rhodoc/ruber/r_ruber77.html
IEGM 84	Sandy soil, Gomel region, Belarus	http://www.iegmcol.ru/strains/rhodoc/ruber/r_ruber84.html
IEGM 90	Surface water reservoir, the Taimir peninsula, Krasnoyarsk region, Russia	http://www.iegmcol.ru/strains/rhodoc/ruber/r_ruber90.html
IEGM 93	Sandy soil, Irkutsk region, Russia	http://www.iegmcol.ru/strains/rhodoc/ruber/r_ruber93.html
IEGM 172	Stratal water, oilfield, Perm krai, Russia	http://www.iegmcol.ru/strains/rhodoc/ruber/r_ruber172.html
IEGM 219	Water, the Upper Ilitch river, Russia	http://www.iegmcol.ru/strains/rhodoc/ruber/r_ruber219.html

R. ruber		
IEGM 223	Soil, Polasna oil-extracting enterprise, Perm krai, Russia	http://www.iegmcol.ru/strains/rhodoc/ruber/r_ruber223.html
IEGM 224	Soil, Polasna oil-extracting enterprise, Perm krai, Russia	http://www.iegmcol.ru/strains/rhodoc/ruber/r_ruber224.html
IEGM 225	Soil, Polasna oil-extracting enterprise, Perm krai, Russia	http://www.iegmcol.ru/strains/rhodoc/ruber/r_ruber225.html, GenBank#KJ442849
IEGM 231	Water, spring, Olkhovski oil-extracting enterprise, Perm krai, Russia	http://www.iegmcol.ru/strains/rhodoc/ruber/r_ruber231.html, DDBJ/ENA/GenBank acc. no CCSD01000001-CCSD01000115
IEGM 233	Soil, Polasna oil-extracting enterprise, Perm krai, Russia	http://www.iegmcol.ru/strains/rhodoc/ruber/r_ruber233.html, GenBank#KJ442850
IEGM 235	Snow, Polasna oil-extracting enterprise, Perm krai, Russia	http://www.iegmcol.ru/strains/rhodoc/ruber/r_ruber235.html, GenBank#KJ442851
IEGM 236	Soil, Polasna oil-extracting enterprise, Perm krai, Russia	http://www.iegmcol.ru/strains/rhodoc/ruber/r_ruber236.html
IEGM 238	Sandy soil, Gomel region, Belarus	http://www.iegmcol.ru/strains/rhodoc/ruber/r_ruber238.html
IEGM 241	Chalk rock (depth 80 m), Gomel region, Belarus	http://www.iegmcol.ru/strains/rhodoc/ruber/r_ruber241.html
IEGM 323	Turf soil, Oktyabrski district, Sverdlovsk region, Russia	http://www.iegmcol.ru/strains/rhodoc/ruber/r_ruber323.html
IEGM 325	Oil-polluted water, Bistrinskoe oilfield, Tyumen region, Russia	http://www.iegmcol.ru/strains/rhodoc/ruber/r_ruber325.html
IEGM 326	Turf soil, oil-gas field, Perm krai, Russia	http://www.iegmcol.ru/strains/rhodoc/ruber/r_ruber326.html, GenBank#MG637021
IEGM 327	Turf soil, oil-gas field, Perm krai, Russia	http://www.iegmcol.ru/strains/rhodoc/ruber/r_ruber327.html, GenBank#KJ442854
IEGM 328	Water, well, oil-gas field, Perm krai, Russia	http://www.iegmcol.ru/strains/rhodoc/ruber/r_ruber328.html
IEGM 334	Sand rock from the depth 6 m, Belarus	http://www.iegmcol.ru/strains/rhodoc/ruber/r_ruber334.html
IEGM 342	Underground water, oilfield, Perm krai, Russia	http://www.iegmcol.ru/strains/rhodoc/ruber/r_ruber342.html, GenBank#KY174957
IEGM 381	Water, the Baykal Lake, Irkutsk region, Russia	http://www.iegmcol.ru/strains/rhodoc/ruber/r_ruber381.html
IEGM 385	Bottomset beds, the Baykal Lake, Irkutsk region, Russia	http://www.iegmcol.ru/strains/rhodoc/ruber/r_ruber385.html

R. ruber		
IEGM 436	Soil, bank of the Pechjora river, Russia	http://www.iegmcol.ru/strains/rhodoc/ruber/r_ruber436.html
IEGM 438	Oil-polluted water, oil-extracting enterprise, Perm krai, Russia	http://www.iegmcol.ru/strains/rhodoc/ruber/r_ruber438.html
IEGM 440	Oil-polluted sand, Belarus	http://www.iegmcol.ru/strains/rhodoc/ruber/r_ruber440.html
IEGM 443	No information	http://www.iegmcol.ru/strains/rhodoc/ruber/r_ruber443.html
Rhodococcus sp.		
IEGM 27	Oil-polluted soil, oil-gas field, Poltava region, Ukraine	http://www.iegmcol.ru/strains/rhodoc/sp/r_sp27.html
IEGM 61	Oil-polluted soil, Ukraine	http://www.iegmcol.ru/strains/rhodoc/sp/r_sp61.html
IEGM 69	Soil, Polasna oil-extracting enterprise, Perm krai, Russia	http://www.iegmcol.ru/strains/rhodoc/sp/r_sp69.html
IEGM 1276	Oil slime, Udmurt Republic, Russia	http://www.iegmcol.ru/strains/rhodoc/sp/r_sp1276.html

For some strains, the species level has status “failed” that is related with current changes in taxonomy and defining of borders for closely related species.

Table S2 – *Rhodococcus* strains selected for adhesion and biodegradation experiments

Strain	Isolation source	Properties
R. erythropolis IEGM 212	Sewage, Kharbin, China	Uses hydrocarbons as a sole carbon source; resistant to Pb^{2+} (20.0 mm), Cr^{6+} (10.0 mm), Cu^{2+} (5.0 mm)
R. erythropolis IEGM 266	Oil-polluted soil, oil-extracting enterprise, Perm region, Russia	Uses hydrocarbons and crude oil as a sole carbon source (95); resistant to Cr^{6+} (20.0 mm), VO^{2+} (12.5 mm), VO_4^{3-} (50.0 mm), VO_3^- (>250.0 mm)
R. opacus IEGM 57	Oil-polluted soil, Ukraine	Uses *n*-hexadecane and crude oil as a sole carbon source; resistant to Cr^{6+} (5.0 mm), VO_4^{3-} (25.0 mm), VO_3^- (>250.0 mm)
R. opacus IEGM 262	Soil, lavsan (polyether fibre) production, Belarus	*Uses* *n*-hexadecane, *n*-docosane, *n*-hexacosane, *n*-octacosane, *n*-nonacosane, *n*-hentriacontane, anthracene, and phenanthrene as a sole carbon and energy source; resistant to Pb^{2+} (5.0 mm)
R. opacus IEGM 717	Soil, UK	Uses *n*-hexadecane as a sole carbon source; resistant to VO_4^{3-} (12.5 mm), VO_3^- (>250.0 mm); accumulates nickel
R. qingshengii IEGM 267	Oil-polluted soil, oil-extracting enterprise, Perm region, Russia	Uses hydrocarbons and crude oil as a sole carbon source; resistant to Cr^{6+} (5.0 mm), VO^{2+} (12.5 mm), VO_4^{3-} (50.0 mm), VO_3^- (250.0 mm)
R. rhodochrous IEGM 63	Oil-polluted soil, Ukraine	Uses hydrocarbons as a sole carbon source; accumulates cesium ions; resistant to Cr^{6+} (5.0 mm), VO^{2+} (12.5 mm), VO_4^{3-} (50.0 mm), VO_3^- (>250.0 mm)
R. rhodochrous IEGM 64	Oil-polluted soil, Ukraine	Uses hydrocarbons as a sole carbon source; resistant to Cr^{6+} (5.0 mm), VO^{2+} (12.5 mm), VO_4^{3-} (50.0 mm), VO_3^- (>250.0 mm), accumulates molybdenum and nickel
R. rhodochrous IEGM 646	Water, Berezniki, Perm region, Russia	Uses hydrocarbons as a sole carbon source; resistant to Cr^{6+} (5.0 mm), VO^{2+} (12.5 mm), VO_4^{3-} (100.0 mm), VO_3^- (>250.0 mm),

		accumulates molybdenum and nickel
R. ruber IEGM 219	Water, the Upper Ilitch river, Russia	Uses propane, *n*-butane (95) and liquid *n*-alkanes (C_5-C_7, C_{11}-C_{16}); resistant to Pb^{2+} (5.0 mm)
R. ruber IEGM 241	Chalk rock (depth 80 m), Gomel region, Belarus	Uses propane, *n*-butane and liquid *n*-alkanes (C_5-C_{16}) as a sole carbon source; accumulates cesium ions; resistant to Cr^{6+} (10.0 mm), Cu^{2+}, Ni^{2+}, Pb^{2+} (5.0 mm)
R. ruber IEGM 328	Water, well, oil-gas field, Perm region, Russia	Uses propane and *n*-butane as a sole carbon source; resistant to Pb^{2+} (5.0 mm)

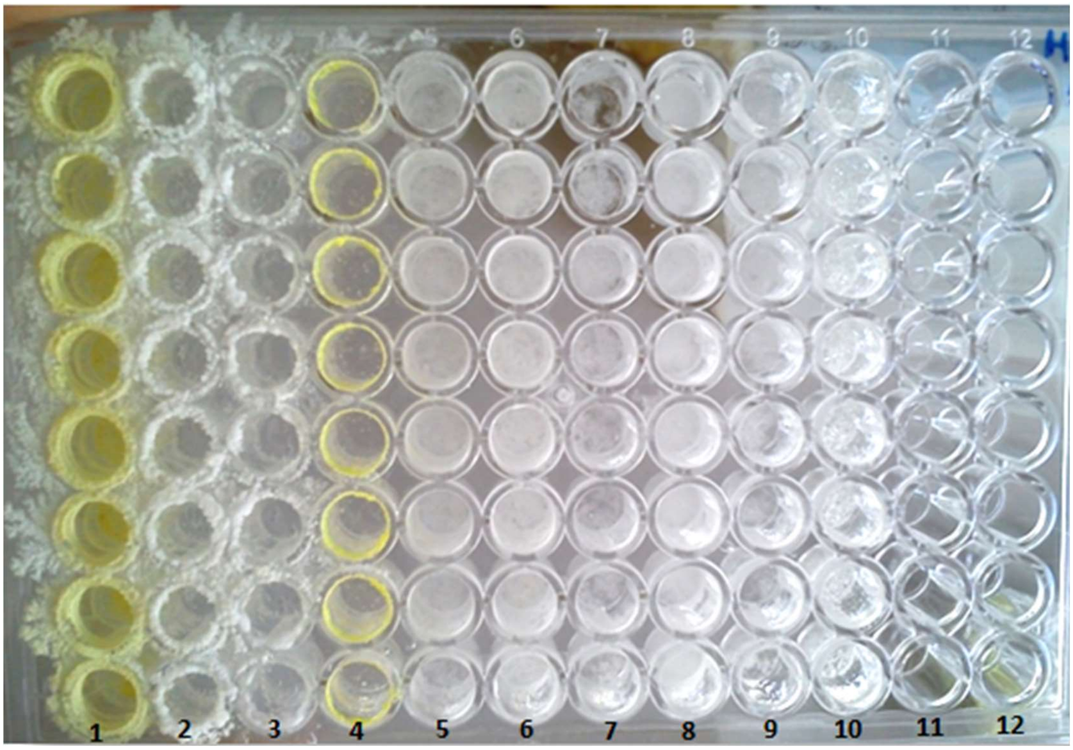

Figure S1 – **The 96-well polystyrene microplate modified using hydrocarbons**

Hydrocarbons: 1 – benzo[a]pyrene, 2 – phenanthrene, 3 – naphthalene, 4 – benzo[a]anthracene, 5 – *n*-octacosane, 6 – *n*-docosane, 7 – *n*-nonacosane, 8 – *n*-hexacosane, 9 – *n*-hentriacontane, 10 – anthracene, 11, 12 – control (without hydrocarbons).

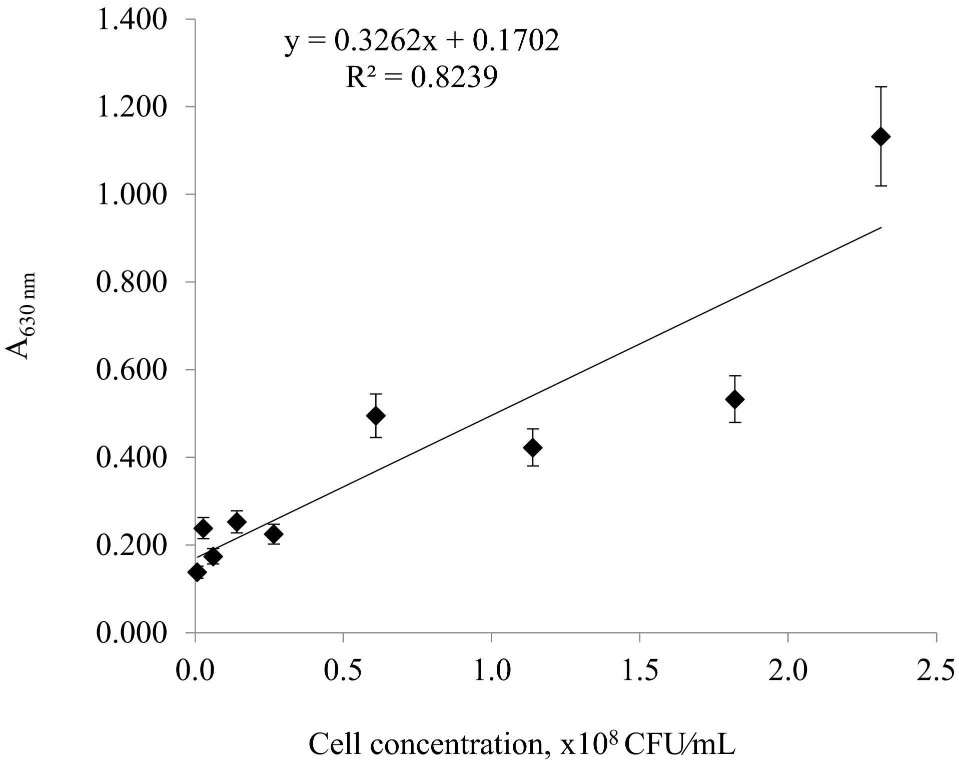

Figure S2 – **Calibration curve between concentration of *Rhodococcus* cells and $A_{630\ nm}$ after staining of cells with crystal violet**

Protocol for calibration: Cells were grown in 250-mL Erlenmeyer flasks with 100 mL LB at 160 rpm and 28 °C for 28–30 h (until the late exponential growth phase), washed twice with 0.5% NaCl, resuspended in 0.5% NaCl and diluted in the range of concentrations between 0 and 2.5×10^8 CFU/mL in increments $0.5\cdot10^8$ and $0.1\cdot10^8$ CFU/mL. For each suspension, a precise number of cells (CFU/mL) on nutrient agar was determined. Suspensions (1 mL) were then centrifuged; supernatant was removed; 1 mL of 1% (w/w) crystal violet was added; cells were resuspended in the dye solution with thorough mixing to obtain a homogenous suspension and left at room temperature for 20 min for staining. Stained cells were centrifuged; supernatant was removed; cells were washed twice with 1 mL 0.5% NaCl (each time cells were centrifuged and resuspended to a homogenous suspension); and the crystal violet was extracted from cells with 1 mL of acetone/ethanol mixture (1:4, v/v) at 160 rpm and room temperature for 5 min. The extract was transferred into polystyrene microplates (200 µL per well), and absorbance at 630 nm was measured with a Multiscan Ascent photometer (Thermo Electron Corporation, Finland). Three replicates of suspensions were used for each cell concentration. A calibration curve was built using the obtained values of $A_{630\ nm}$ and CFU/mL.

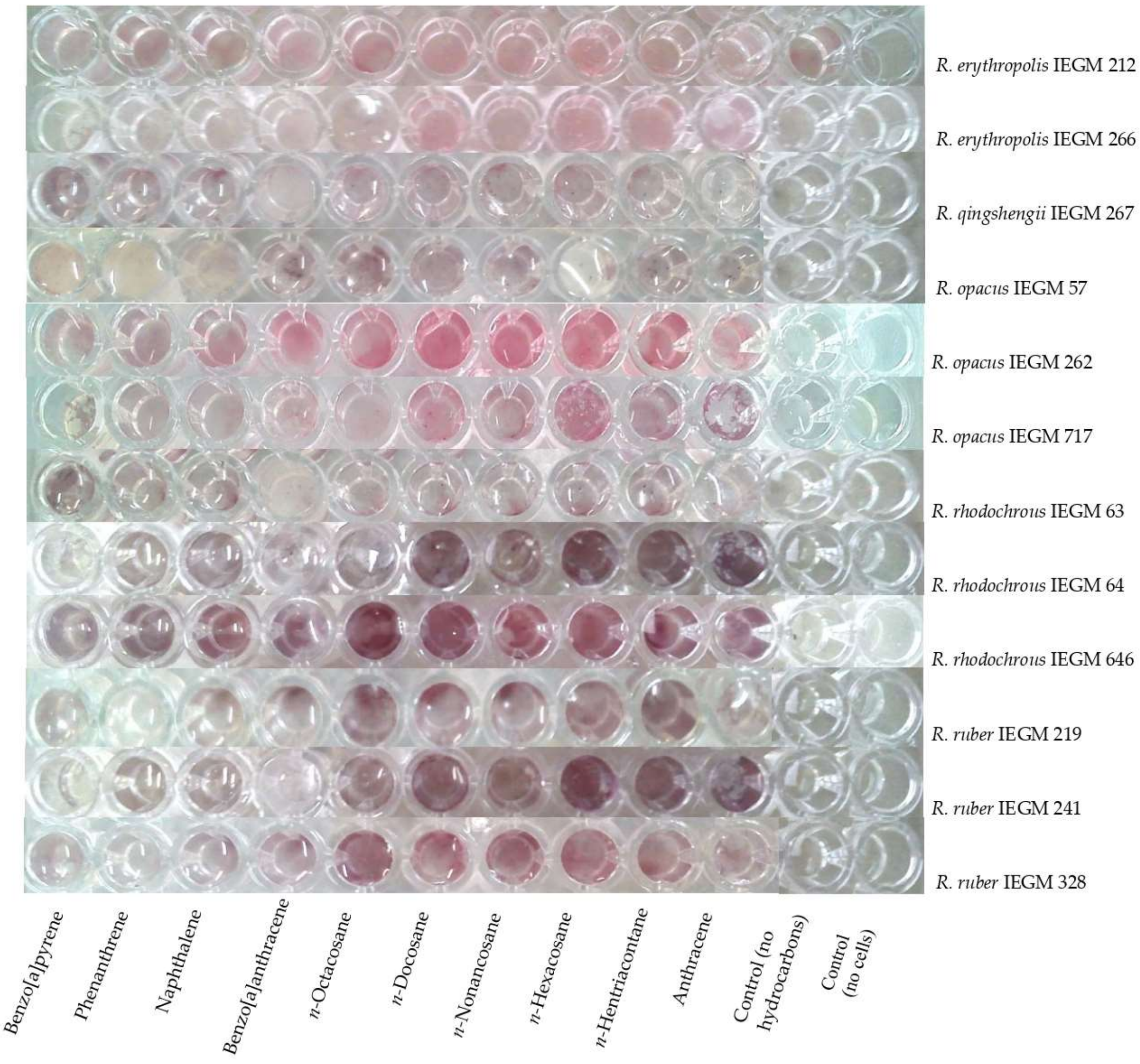

Figure S3 – **Growth of *Rhodococcus* cells in the presence of 0.2 mM individual C22–C31 *n*-alkanes or PAHs detected with INT staining after incubation for 72 h**

Control (no growth) – medium with cells and without hydrocarbons. Control (no cells) – medium with hydrocarbons and without cells.

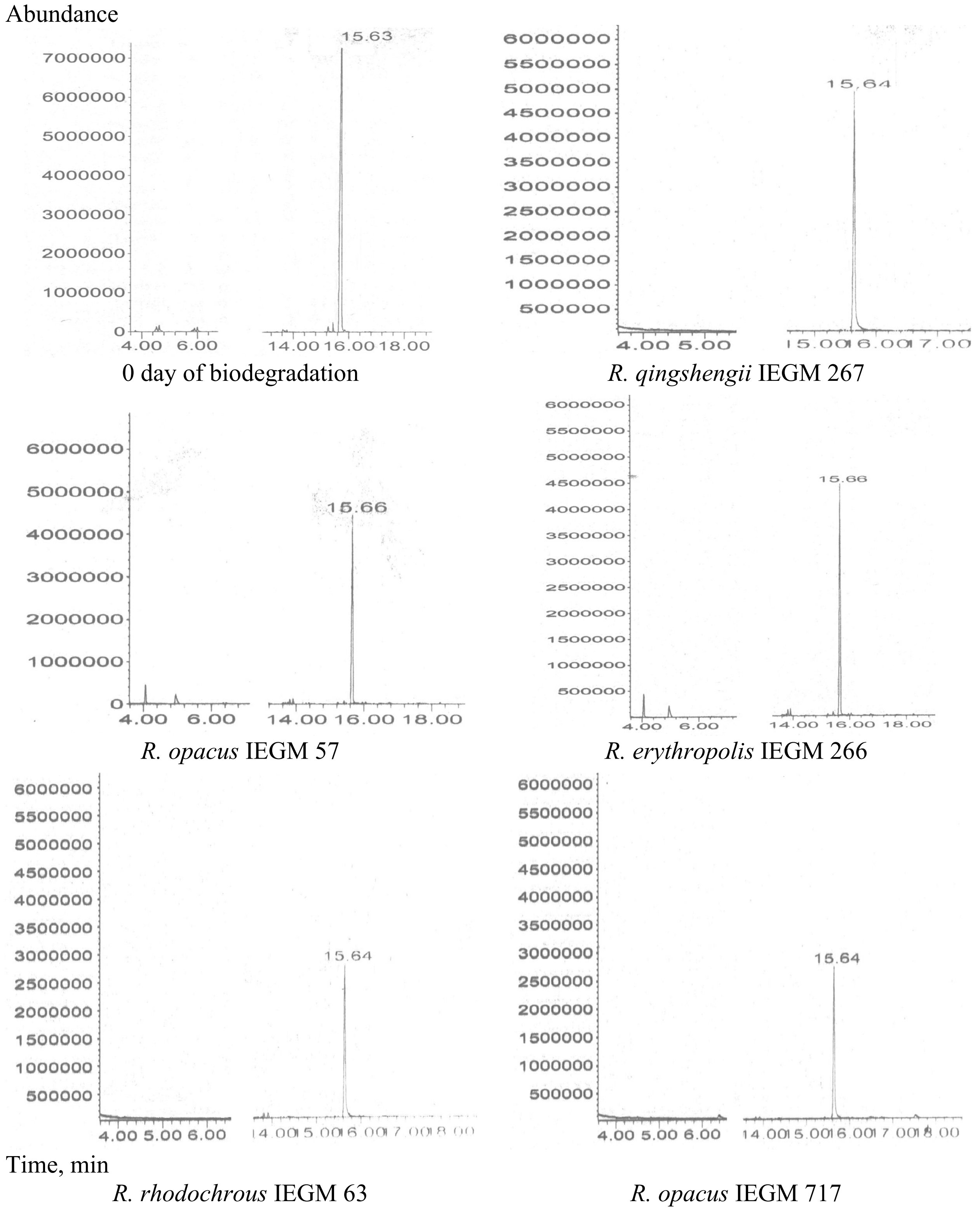

Figure S4 – **GC-MS chromatograms of residual anthracene** (retention time 15.63–15.66 min) **after 9 days of biodegradation by *Rhodococcus* cells**

Typical (average) chromatograms are shown. Dilution factor was same for all presented chromatograms. Total areas of peaks were used for calculation of residual anthracene concentration. The initial (0 day of biodegradation) concentration of anthracene in samples was 2.00±0.17 mg/mL.

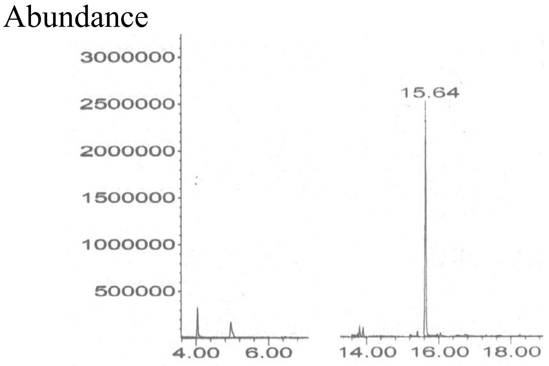

R. ruber IEGM 328

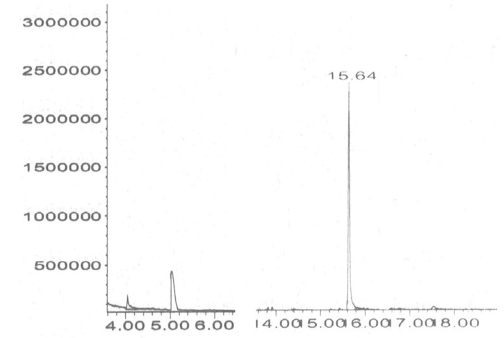

R. ruber IEGM 219

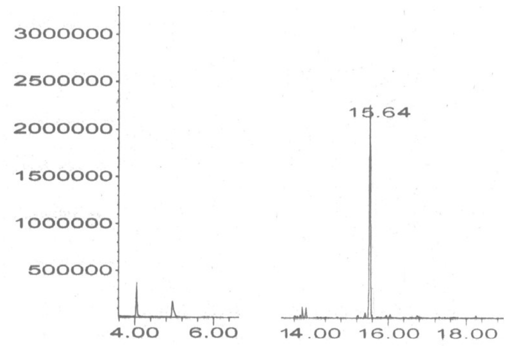

R. rhodochrous IEGM 646

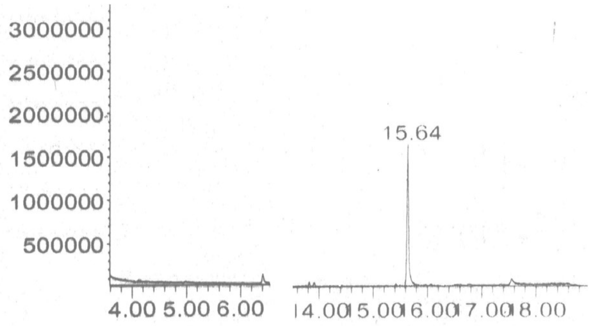

R. ruber IEGM 241

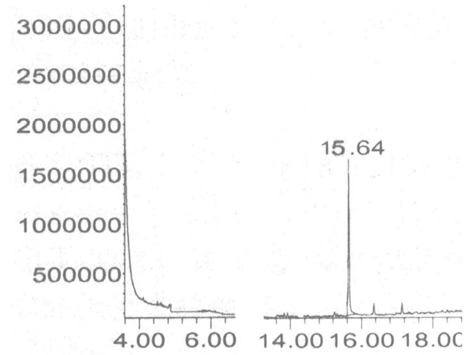

R. rhodochrous IEGM 64

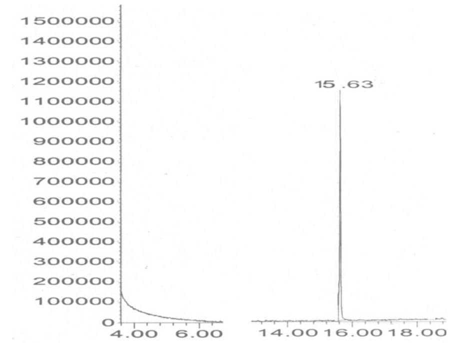

R. erythropolis IEGM 212

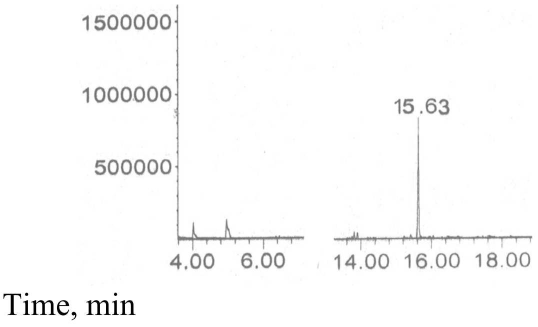

R. opacus IEGM 262

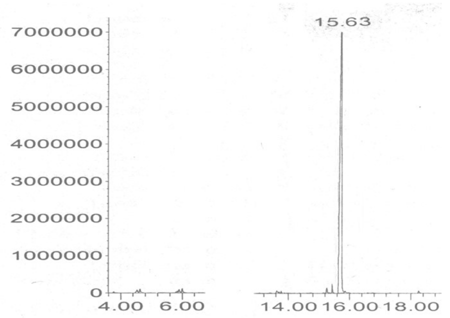

No cells (abiotic control)

Figure S4 – **GC-MS chromatograms of residual anthracene** (retention time 15.63–15.66 min) **after 9 days of biodegradation by *Rhodococcus* cells**

Typical (average) chromatograms are shown. Dilution factor was same for all presented chromatograms. Total areas of peaks were used for calculation of residual anthracene concentration. The initial (0 day of biodegradation) concentration of anthracene in samples was 2.00±0.17 mg/mL.

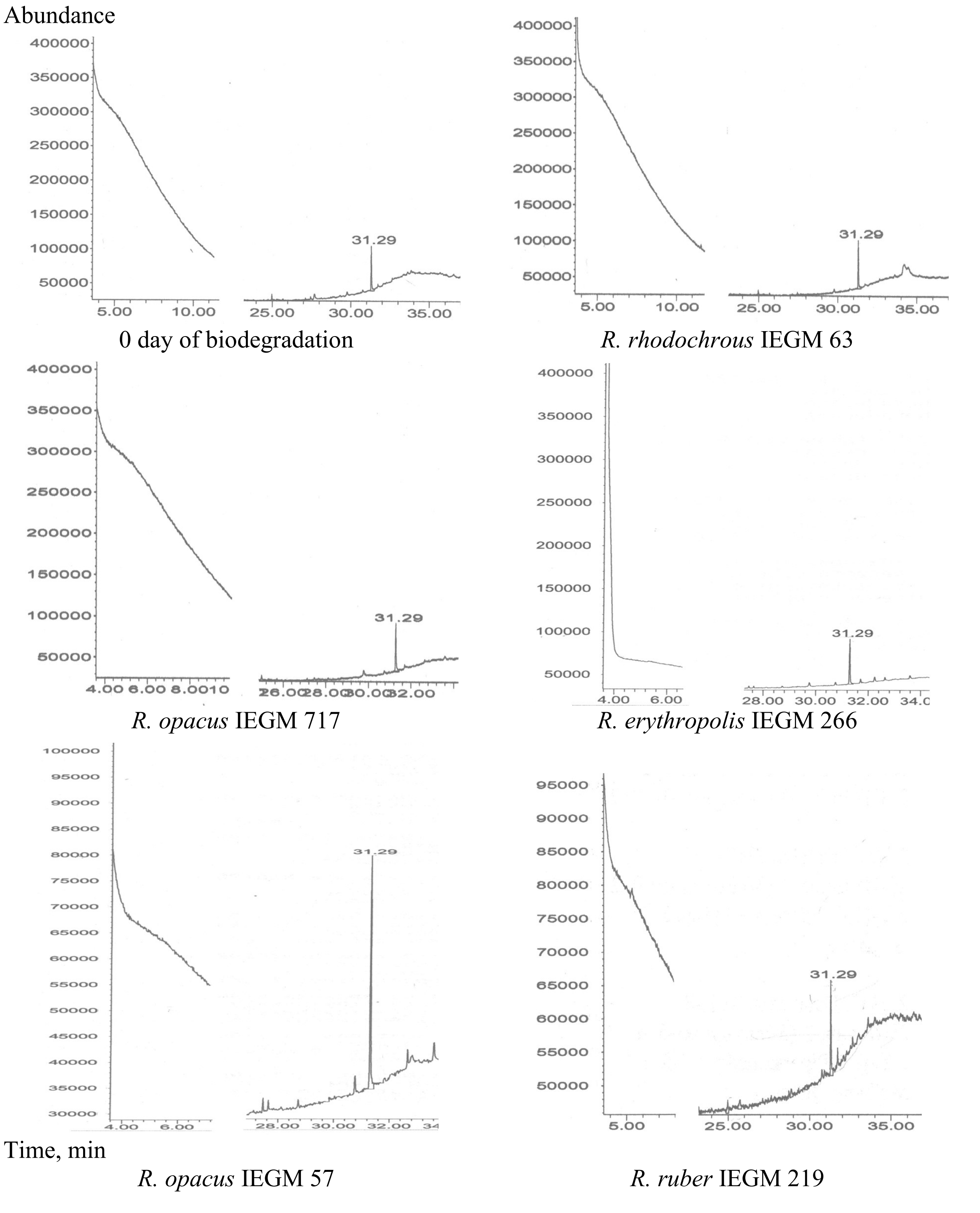

Figure S5 – **GC-MS chromatograms of residual *n*-hexacosane** (retention time 31.27–31.29 min) **after 9 days of biodegradation by *Rhodococcus* cells**

Typical (average) chromatograms are shown. Dilution factor was same for all presented chromatograms. Total areas of peaks were used for calculation of residual *n*-hexacosane concentration. The initial (0 day of biodegradation) concentration of *n*-hexacosane in samples was 2.05±0.18 mg/mL.

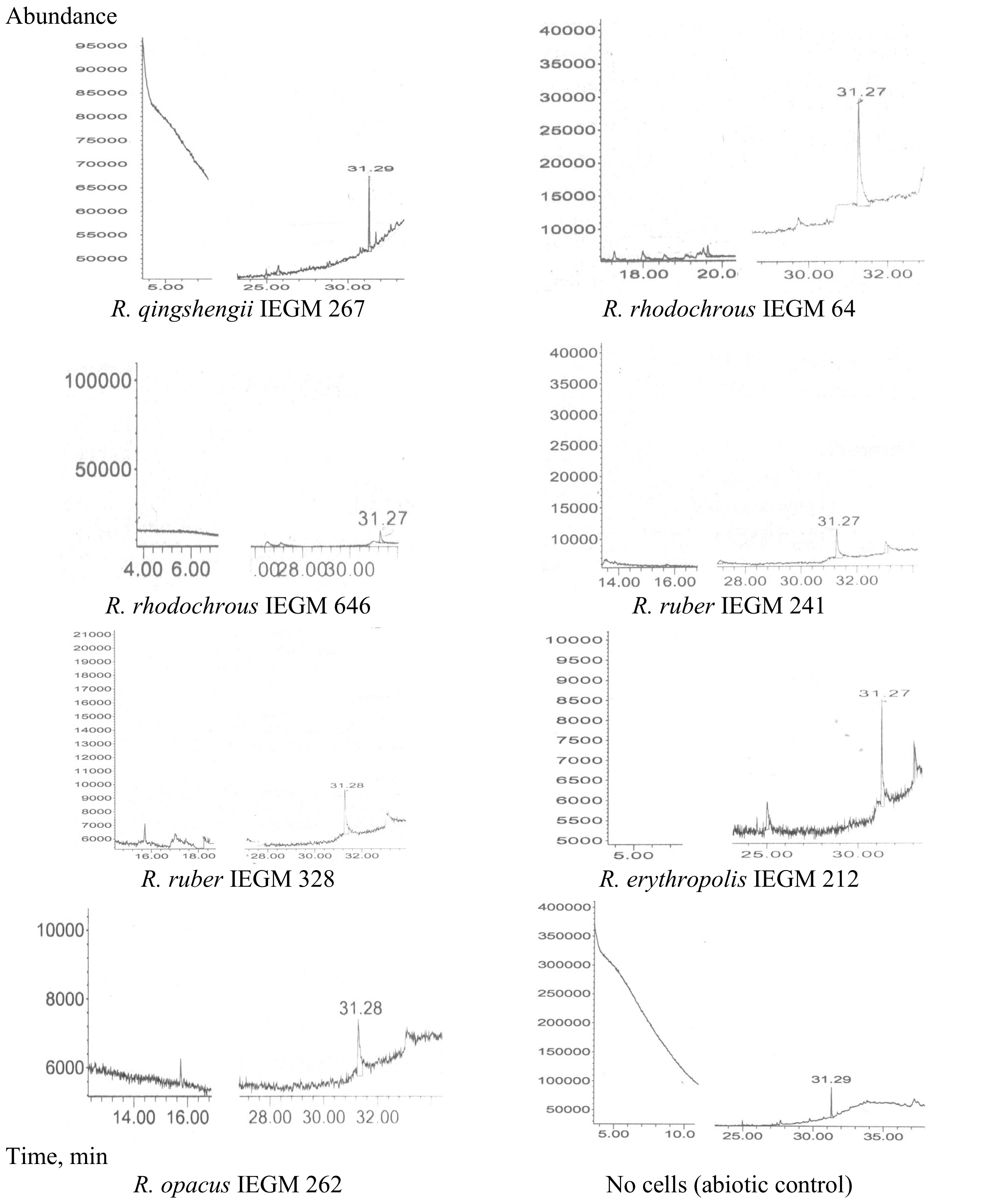

Figure S5 – **GC-MS chromatograms of residual *n*-hexacosane** (retention time 31.27–31.29 min) **after 9 days of biodegradation by *Rhodococcus* cells**

Typical (average) chromatograms are shown. Dilution factor was same for all presented chromatograms. Total areas of peaks were used for calculation of residual *n*-hexacosane concentration. The initial (0 day of biodegradation) concentration of *n*-hexacosane in samples was 2.05±0.18 mg/mL.

Table S3 – Correlations between adhesive activities of *Rhodococcus* strains[1] towards C22–C31 *n*-alkanes and PAHs and physicochemical properties of these hydrocarbons

Property of a hydrocarbon substrate	$R_{Spearman}$	*p*-value
Water solubility	≤ 0.6	≥ 0.08
$logP_{O/W}$	≤ 0.2	≥ 0.10
Molecular weight	≤ 0.2	≥ 0.10
Length of chain (for C22–C31 *n*-alkanes)	≤ 0.6	≥ 0.06
Number of condensed benzene rings (for PAHs)	≤ 0.2	≥ 0.08

[1]Number of strains studied *n* = 12 (see Table S2).

Table S4 – Medians of adhesion force and elastic modulus on the surface of *Rhodococcus* cells

Strain	Adhesion force (F_a), nN	Elastic modulus (E), MPa
R. erythropolis IEGM 212	7.1	1.4
R. erythropolis IEGM 266	4.4	7.4
R. opacus IEGM 57	1.4	1.8
R. opacus IEGM 262	1.0	27.5
R. opacus IEGM 717	1.4	3.6
R. qingshengii IEGM 267	0.2	0.2
R. rhodochrous IEGM 63	1.8	6.0
R. rhodochrous IEGM 64	3.2	12.0
R. rhodochrous IEGM 646	0.4	0.2
R. ruber IEGM 219	1.1	7.1
R. ruber IEGM 241	1.7	8.1
R. ruber IEGM 328	2.8	31.8

Table S5 – Correlation coefficients between adhesion force, elastic modulus and adhesive activity of *Rhodococcus* bacteria

Adhesive activity towards	Adhesion force (F_a)		Elastic modulus (E)	
	$R_{Spearman}$	*p*-value	$R_{Spearman}$	*p*-value
Polystyrene	0.2	0.60	0.4	0.24
n-Docosane	0.2	0.53	0.5	0.12
n-Hexacosane	0.1	0.77	0.5	0.07
n-Octacosane	0.1	0.70	0.5	0.09
n-Nonacosane	0.0	0.98	0.6	0.05
n-Hentriacontane	0.3	0.34	0.4	0.18
Naphthalene	**0.6**	**0.03**	0.1	0.86
Anthracene	0.0	0.96	0.5	0.10
Phenanthrene	0.5	0.10	0.4	0.26
Benzo[a]pyrene	0.2	0.59	0.5	0.13
Benzo[a]anthracene	0.6	0.05	0.3	0.35

Table S6 – Correlation coefficients between adhesive activities of *Rhodococcus* strains[1] and physicochemical properties of cells

Adhesion substrate	Hydrophobicity of cells				Zeta potential of cells	
	MATH		SAT			
	$R_{Spearman}$	*p*-value	$R_{Spearman}$	*p*-value	$R_{Spearman}$	*p*-value
n-Docosane	0.16	≥ 0.05	–0.30	≥ 0.05	0.40	≥ 0.05
n-Hexacosane	0.29	≥ 0.05	–0.35	≥ 0.05	0.15	≥ 0.05
n-Octacosane	0.21	≥ 0.05	–0.35	≥ 0.05	0.34	≥ 0.05
n-Nonacosane	0.15	≥ 0.05	–0.32	≥ 0.05	0.35	≥ 0.05
n-Hentriacontane	0.04	≥ 0.05	–0.28	≥ 0.05	0.48	≥ 0.05
Naphthalene	0.21	≥ 0.05	–0.17	≥ 0.05	–0.19	≥ 0.05
Anthracene	0.24	≥ 0.05	–0.24	≥ 0.05	0.07	≥ 0.05
Phenanthrene	–0.17	≥ 0.05	–0.13	≥ 0.05	0.27	≥ 0.05
Benzo[a]pyrene	0.07	≥ 0.05	–0.36	≥ 0.05	0.47	≥ 0.05
Benzo[a]anthracene	0.13	≥ 0.05	–0.24	≥ 0.05	0.18	≥ 0.05
Polystyrene	0.28	≥ 0.05	–0.57	≥ 0.05	0.15	≥ 0.05

[1]Number of the strains studied n = 12 (see Table S2).

Table S7 – Adhesion of *Rhodococcus* cells to MATS substrates, %

Strain	MATS substrate					
	n-Hexane	*n*-Decane	*n*-Hexadecane	Diethyl ester	Ethyl acetate	Chloroform
R. erythropolis IEGM 212	71 ± 9	68 ± 25	24 ± 17	66 ± 18	5 ± 4	83 ± 6
R. erythropolis IEGM 266	38 ± 1	34 ± 8	29 ± 4	63 ± 1	85 ± 4	11 ± 8
R. opacus IEGM 57	89 ± 14	94 ± 5	99 ± 1	33 ± 7	100 ± 0	90 ± 9
R. opacus IEGM 262	0 ± 0	0 ± 0	62 ± 12	75 ± 14	91 ± 15	65 ± 7
R. opacus IEGM 717	39 ± 13	39 ± 13	32 ± 13	77 ± 10	36 ± 6	55 ± 18
R. qingshengii IEGM 267	54 ± 4	69 ± 8	55 ± 6	96 ± 1	78 ± 18	82 ± 2
R. rhodochrous IEGM 63	33 ± 4	71 ± 4	25 ± 14	79 ± 7	0 ± 0	77 ± 9
R. rhodochrous IEGM 64	89 ± 5	85 ± 17	87 ± 3	93 ± 6	99 ± 2	87 ± 1
R. rhodochrous IEGM 646	32 ± 3	42 ± 9	38 ± 1	71 ± 11	14 ± 3	61 ± 4
R. ruber IEGM 219	69 ± 6	73 ± 23	75 ± 2	84 ± 12	40 ± 20	77 ± 1
R. ruber IEGM 241	49 ± 23	59 ± 15	60 ± 1	79 ± 23	86 ± 13	72 ± 4
R. ruber IEGM 328	36 ± 2	45 ± 2	37 ± 2	69 ± 3	61 ± 13	42 ± 4
Correlations with adhesive activities towards solid hydrocarbons and polystyrene	$R_{Spearman} \leq 0.56$[1], $p \geq 0.05$	$R_{Spearman} \leq 0.54$, $p \geq 0.05$	$R_{Spearman} \leq 0.36$, $p \geq 0.05$	$R_{Spearman} \leq 0.39$, $p \geq 0.05$	$R_{Spearman} \leq 0.36$, $p \geq 0.05$	$R_{Spearman} \leq 0.41$, $p \geq 0.05$

Number of the strains studied n = 12 (see Table S2). [1]Absolute values for the correlation coefficients are shown.
